# Lower-Limb Motor Function, Mobility, Balance, Falls and Intervention Effects in Inclusion Body Myositis: A Systematic Review

**DOI:** 10.3390/jcm15166304

**Published:** 2026-08-14

**Authors:** Dhruv Nandakumar, Manuel Lubinus, Woohyoung Jeon

**Affiliations:** 1 Myositis Support and Understanding Association (MSU), Lincoln, DE 19960, USA; mlubinus@understandingmyositis.org; 2 Department of Health and Kinesiology, The University of Texas at Tyler, Tyler, TX 75799, USA; wjeon@uttyler.edu

**Keywords:** inclusion body myositis, sporadic inclusion body myositis, quadriceps strength, gait, falls, postural balance, peripheral neuropathy, rehabilitation, resistance training, systematic review

## Abstract

**Background/Objectives:** Inclusion body myositis (IBM) causes progressive, quadriceps-predominant weakness that impairs mobility and increases fall risk, yet outcomes most relevant to independence remain unsynthesized for IBM. This review compiles direct IBM evidence across five domains (A1–A5): natural history, motor performance, falls and balance, sensory/peripheral-nerve function, and interventions. **Methods:** Eight databases and two trial registers were searched without date or language limits. Eligible studies enrolled adults with IBM based on recognized criteria reporting lower-limb strength or function, gait, transitional tasks, balance, falls, or intervention outcomes; mixed-myopathy cohorts required extractable IBM-specific data. Two reviewers independently screened, extracted, and appraised risk of bias, following PRISMA 2020. **Results:** Sixty-four studies were included; per-domain totals (A1: 16, A2: 9, A3: 6, A4: 2, and A5: 38) exceed 64 because studies may span domains. Quadriceps strength was the most sensitive progression marker, detected earlier by quantitative testing. Falls were near-universal and insufficiently managed. No drug showed convincing functional benefit in controlled trials, whereas exercise and orthotic/robotic assistance appeared to be safe in small studies. Sensory and peripheral-nerve dysfunction were common, but proprioceptive acuity and postural balance were unmeasured. **Conclusions:** IBM progression is best measured by quantitative quadriceps strength and function. Intervention evidence derives largely from small, uncontrolled and neutral trials. Primary myopathy is likely the principal driver of decline, but its downstream consequences—for balance, proprioception, and falls—remain underexplored and are the priority for future study.

## 1. Introduction

Inclusion body myositis (IBM) represents the most prevalent idiopathic inflammatory myopathy in adults over 50 years of age (Meyer et al. [1]; Callan et al. [2]). Clinically, IBM is characterized by slowly progressive, asymmetric muscle weakness, with early and disproportionate involvement of the quadriceps and long-finger flexors. Pathologically, it is defined by endomysial inflammation, rimmed vacuoles, and protein aggregation. Quadriceps weakness leads to knee instability, impaired transfers and gait, and a substantial risk of falls and fall-related injuries. Currently, no disease-modifying therapy of established benefit exists, and management remains primarily supportive and rehabilitative.

Although the literature on inclusion body myositis is expanding, evidence regarding outcomes that most influence independence—such as lower-limb strength and its decline, gait and transitional-task performance, balance and falls, and the effects of interventions on these domains—has not been synthesized in an IBM-specific manner. Existing systematic reviews have primarily focused on pharmacological treatment (Saltychev et al. [3]; Rose et al. [4]; Naddaf et al. [5]), for which no agent has shown a clear functional benefit, or on diagnosis and pathology, while balance and rehabilitation have only recently been reviewed and in isolation (Jeon et al. [6]); broader reviews of myopathy, falls, or rehabilitation have tended to group IBM with other conditions. To date, no review has specifically synthesized the progression of lower-limb strength, gait and transfer performance, falls, and the effects of interventions on these outcomes in IBM. Previous syntheses have also tended to include indirect (non-IBM) and mechanistic studies in the evidence base and to count publications rather than distinct studies. The present review addresses these limitations by restricting the evidence to primary research involving individuals with IBM that reports outcomes within a defined lower-limb and mobility scope, mapping each report to its parent study, and reserving the indirect and mechanistic literature for the Section 4. This review is organized around five key questions: A1, natural history and progression; A2, motor performance; A3, falls and balance; A4, lower-limb sensory, proprioceptive, and peripheral nerve function; and A5, intervention effects.

## 2. Materials and Methods

### 2.1. Design and Reporting

This systematic review examined direct human evidence in inclusion body myositis (IBM) and was organized around five prespecified review questions: natural history and disease progression (A1), motor performance in gait and transitional tasks (A2), falls and balance (A3), lower-limb sensory/proprioceptive and peripheral-nerve function (A4), and intervention effects (A5). The review defined its objectives, eligibility criteria, search strategy, data items, and analysis approach at the outset, although these were not lodged in a public register or a formally dated protocol document. Reporting adhered to the PRISMA 2020 [7] statement, and synthesis without meta-analysis was conducted according to SWiM [8] (Synthesis Without Meta-analysis) guidance. Indirect (non-IBM), animal, and secondary (narrative/systematic-review) sources were excluded from the primary evidence base and referenced only in the Section 4 to inform hypothesis development. The completed PRISMA 2020 checklist is provided in Supplementary File S2.

### 2.2. Protocol and Registration

This review was not prospectively registered on a public register (e.g., PROSPERO). To mitigate the increased risk of post hoc decisions resulting from the absence of prospective registration, and because no formal dated protocol document was prepared, the eligibility rules, procedures for handling mixed cohorts, risk-of-bias instruments, and certainty framework are reported here in full; we therefore do not imply that every decision was definitively prespecified, and the applied rules are reported so the review can be reproduced and audited. This limitation is explicitly acknowledged in Section 4. In addition, the present version incorporates substantive post hoc modifications made in response to peer-review comments—including expansion and re-running of the database search (with removal of outcome-direction terms and the 20-year date limit), addition of the lower-limb sensory and peripheral-nerve arm (A4), and reclassification of previously-counted studies as clearly labeled Discussion-only context—none of which were prespecified; these changes are described where they occur, cataloged study-by-study in Supplementary File S6, and acknowledged as a limitation (Section 4.7).

### 2.3. Eligibility Criteria

Population. Adults diagnosed with sporadic IBM according to a recognized, explicitly stated criteria set (e.g., Griggs et al. [9]; ENMC criteria [10]) were included. For each study, the criteria set and diagnostic certainty were documented, and diagnostic heterogeneity across historical and current definitions was treated as a source of clinical heterogeneity. Mixed idiopathic-inflammatory-myopathy cohorts were included only if IBM-specific data were separately reported or could be obtained; studies lacking isolatable IBM-specific data were excluded.

Scope of outcomes. The review focused on lower-limb motor function, mobility, gait, transitional tasks (including sit-to-stand, stand-to-sit, and timed up-and-go), balance, and falls, as well as direct measurement of lower-limb sensory, proprioceptive, or peripheral-nerve function (A4) within the same lower-limb scope. Studies were included only if at least one relevant outcome with extractable data was reported. Reports addressing only isolated finger-flexor/grip strength, dysphagia, biomarker, or diagnostic-utility endpoints were excluded from the evidence base; distal and bulbar features were retained solely as phenotype context. A4 was considered part of lower-limb dysfunction rather than a separate phenotype: only studies directly measuring lower-limb sensory or peripheral-nerve function (such as quantitative sensory testing of the feet or legs, lower-limb somatosensory thresholds, sensory nerve-conduction studies, or lower-limb nerve-fiber pathology) were eligible. Motor electromyography/motor-unit studies and diagnostic-utility-only reports were excluded and referenced as mechanistic context in the Section 4.

Eligible designs. Eligible study designs included randomized controlled trials, non-randomized intervention studies, prospective and retrospective cohort studies, analytical cross-sectional studies, case–control studies, case series, and case reports. Each design was mapped to the review question it could address and to a design-appropriate appraisal instrument (Section 2.7). Conference abstracts were included only as separately tagged gray literature and did not carry the same evidential weight as full reports. Narrative reviews, systematic reviews/meta-analyses, and animal (preclinical) studies were excluded from the primary evidence base; relevant examples were cited only as mechanistic context in the Section 4.

Exclusions. Studies were excluded if IBM-specific data could not be isolated from a mixed cohort, if no relevant lower-limb, mobility, balance, or falls outcome was reported, if the report was a duplicate or companion publication of an already-included study (collapsed to the parent study, Section 2.5), or if the record was a diagnostic/biomarker-only, editorial, or protocol-only document without results. No date or language restrictions were imposed; older natural-history and rehabilitation reports were retained due to the rarity of the condition.

### 2.4. Information Sources and Search

Eight bibliographic databases were searched: MEDLINE/PubMed, Embase, Scopus, Web of Science, Cochrane CENTRAL, CINAHL, SPORTDiscus, and PEDro. Additionally, two trial registries, ClinicalTrials.gov and the WHO ICTRP, were searched to identify completed, unpublished, and ongoing intervention studies. Search strategies combined an IBM population concept with concept blocks for each review question, utilizing both controlled vocabulary (MeSH/Emtree) where appropriate and free-text terms in title, abstract, and keyword fields. Outcome-direction words such as “significant” or “difference” were not used as eligibility filters. Full database-specific strategies, last-searched dates, and reconciliation of companion reports are provided in the appendices. Reference lists of included studies and relevant reviews were screened, and forward-citation tracking was conducted. In response to reviewer feedback that the previous search contained both overly restrictive terminology and date requirements, the search terms were revised—removing outcome-direction language such as “significant” and “difference”—and re-run across all databases on 28 July 2026 with no date restriction applied. Also, in response to reviewer feedback, the number of bibliographic databases queried was increased from the three searched in the previous version (PubMed, Scopus, and Embase) to the eight databases listed above, with the further addition of two trial registries.

### 2.5. Study Selection and De-Duplication

**Figure 1:**
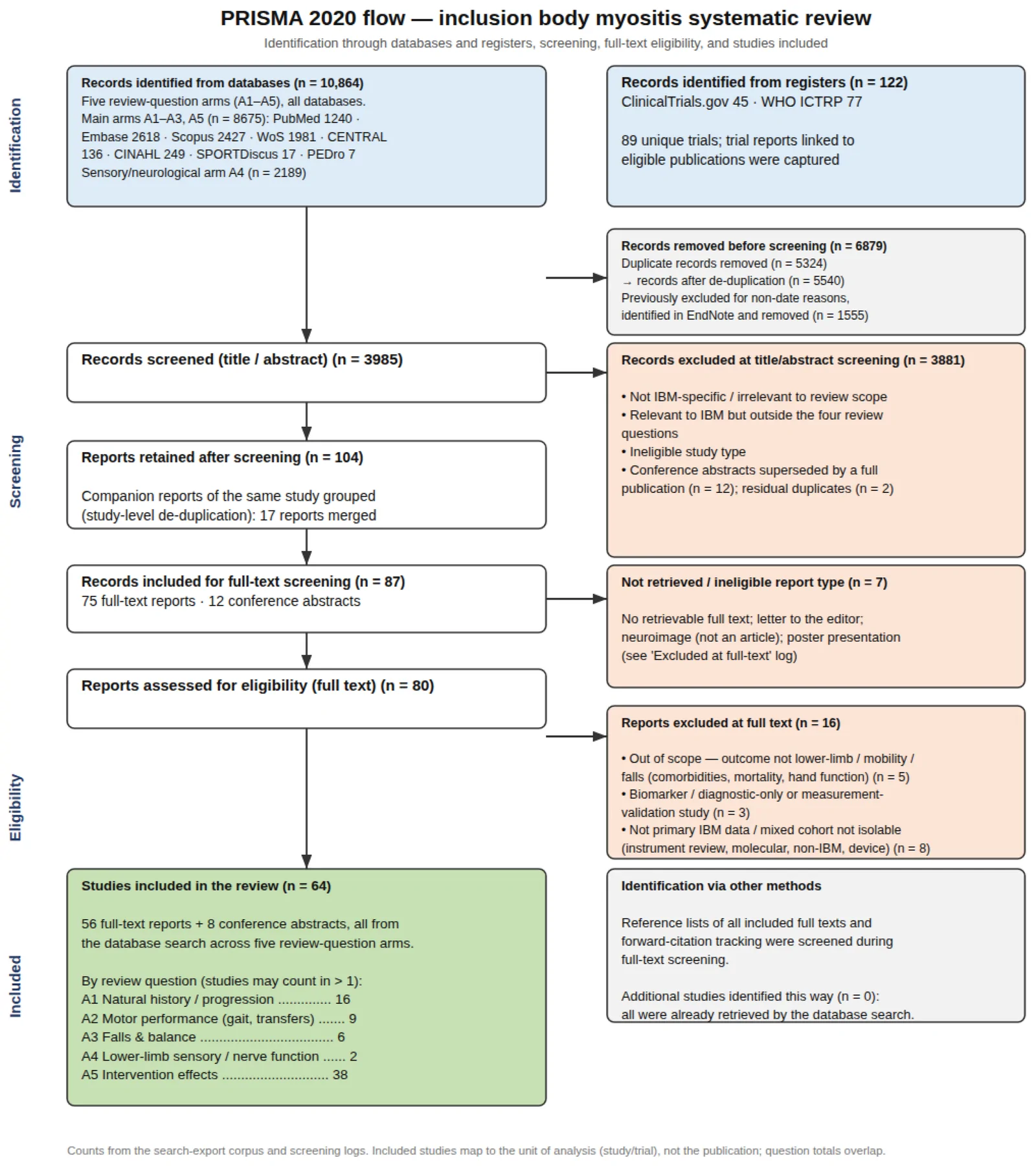
PRISMA 2020 flow diagram of study identification, screening, full-text eligibility, and the studies included in the review.

Records were de-duplicated first at the record level (DOI and normalized title) and subsequently at the study level, with companion reports of the same study or trial explicitly collapsed so that the unit of analysis was the study rather than the publication. This approach prevented a single trial or cohort, such as the blood-flow-restriction trial and its substudy and case reports, or multi-publication pharmacological programs, from being counted more than once. Two reviewers independently screened titles and abstracts, followed by full texts, against the eligibility criteria. Disagreements were resolved by consensus between the two reviewers. The flow of records is presented in a PRISMA 2020 diagram (Figure 1), and a full-text exclusion list with individual reasons (Supplementary File S3) and a study-level de-duplication map linking each companion, secondary, and duplicate report to its parent study (Supplementary File S5) are provided. After the de-duplication stage, 1555 records that had been excluded in the previous version for reasons unrelated to the date limit were removed before title/abstract screening. For the 88 records that reached full-text screening, reviewers were blinded to all bibliographic data (authorship, journal, and year), and the records were presented in randomized order, to mitigate the risk of selection bias. The differences in studies included and excluded between the previous search and the revised search, with reasons, are provided as supplementary material (Supplementary File S6). The search terminology used in the previous version is also reproduced there, with the bracketing and syntax errors identified in peer review corrected.

### 2.6. Data Extraction

Two reviewers independently extracted data using a piloted form and verified entries against the original sources. Extracted items included study design; diagnostic criteria and certainty; participant number, age, sex distribution, disease duration, severity, and ambulatory status; comorbidities and prior or concurrent treatment; muscles assessed and testing methods; gait and transitional-task parameters; for interventions, the intervention and comparator with frequency, intensity, time, type, progression, supervision, and adherence; adverse events and safety; follow-up duration and attrition; prespecified outcomes; numerical effect estimates with measures of uncertainty (confidence intervals) and the numbers analyzed; and funding, commercial involvement, and conflicts of interest. Pharmacological trials and rehabilitation studies were tabulated separately, and unique-participant counts were monitored to account for overlap.

### 2.7. Risk-of-Bias Assessment

Risk of bias was assessed at the outcome level using instruments appropriate for each study design. Randomized trials were evaluated with RoB 2 [11] (five domains, at the level of the primary patient-important outcome); non-randomized intervention studies with ROBINS-I [12] (seven domains); prognostic and natural-history cohorts with QUIPS [13] (six domains); and analytical cross-sectional studies (including A4 sensory-function studies), case series, and case reports with the corresponding JBI [14] critical-appraisal checklists, interpreted at the domain level. Judgments were made at the domain level with written justifications rather than numerical scores, and were made independently by two reviewers, with disagreements resolved through discussion. Conference abstracts were not formally appraised due to insufficient methodological detail to support confident domain-level judgment and were flagged accordingly. SANRA and SYRCLE were not used, as narrative reviews and animal studies were outside the primary evidence base.

### 2.8. Certainty of Evidence (GRADE [15])

Certainty was rated separately for intervention questions and for descriptive or prognostic questions, and ratings were not transferred between these categories. For intervention outcomes (A5), randomized-trial evidence began at high certainty and non-randomized evidence at low certainty, with ratings downgraded for risk of bias, inconsistency, indirectness, imprecision, and publication bias. No body of evidence was upgraded for a “large effect” because no pooled estimate or prespecified large-effect threshold was defined, and the studies employed heterogeneous designs and outcomes. Patient-important benefits and harms were rated independently, and drug and non-drug interventions were separated into distinct rows (drug effects, conventional exercise, aerobic training, blood-flow-restricted training, orthoses, and robotic assistance). For prognostic or natural-history (A1) and falls-burden (A3) outcomes, the GRADE framework for overall prognosis and prognostic factors was used, so that ratings describe certainty in the estimated rate or association rather than a treatment effect. Where no eligible IBM-specific study existed for a concept included in the search (such as measured postural balance/posturography within arm A3 or task-specific or transfer rehabilitation and dedicated balance training within arm A5a), this was reported as a searched-but-empty evidence gap rather than a low-certainty estimate. Proprioceptive, sensory, and neurological function were subsequently searched directly in IBM (Appendix A, arm A4), confirming direct evidence of peripheral-nerve or sensory dysfunction, now synthesized as review question A4 (Section 3.4), while proprioceptive acuity (joint-position sense) and posturography remain unmeasured in IBM and are therefore residual gaps.

### 2.9. Data Synthesis

Due to clinical and methodological heterogeneity and the predominance of small, uncontrolled studies, a structured narrative synthesis was conducted, grouping studies by review question and outcome domain. Meta-analysis was reserved for outcomes with sufficiently homogeneous studies and was not appropriate for any group in this review. In accordance with SWiM, synthesis was based on the direction, magnitude, and precision of numerical effects rather than vote-counting or statistical-significance counts. Sample size and risk of bias informed interpretation, and contradictory findings were explicitly examined. Overlap across the five questions was reconciled in the appendices without inflating the study count.

## 3. Results

Sixty-four studies met eligibility (56 full peer-reviewed reports and eight conference abstracts). Contributions overlap across questions (a study may inform more than one): 16 studies addressed natural history (A1), 9 motor performance (A2), 6 falls and balance (A3), 2 lower-limb sensory/proprioceptive and peripheral-nerve function (A4), and 38 interventions (A5).

### 3.1. Natural History and Disease Progression (A1)

In natural-history cohorts, IBM demonstrates a slow but consistent progression. Quantitative knee-extensor (quadriceps) strength is consistently identified as the most sensitive marker of decline. Allenbach et al. [16] reported that, over nine months, only knee-extension strength changed significantly, and only knee-extension correlated with disease duration, with a quadriceps decline of approximately 13%, while the whole-body composite changed minimally. Longer-term studies support these findings: Hogrel et al. [17] observed a 32% decrease in composite quantitative strength over four years (approximately 9% per year), with knee extension losing about half its value. In contrast, manual muscle testing (MMT) declined by only 1% per year, indicating that quantitative testing was about eight times more sensitive than MMT. Cortese et al. [18] also found the standardized response mean was greatest for quadriceps quantitative strength (27.9% loss over one year) and the IBM Functional Rating Scale (IBM-FRS; 13.8% loss), both surpassing MMT. Cox et al. [19] quantified a 10-year loss of 39% on quantitative testing compared to 29% on MMT, while Sangha et al. [20] reported annual declines of 3.7% (MMT), 3.8% (quantitative), and 6.3% (IBM-FRS), with more rapid early loss. Rose et al. [21] observed a mean 4% composite decline over six months with significant inter-patient variability, and Lindberg et al. [22] reported a natural (untreated) rate of decline near 1% per month, with the fastest progression in the knee extensors.

Longitudinal profiling studies consistently indicate that knee extension and pinch grip decline most rapidly, and that disease progression is faster in men (Lilleker et al. [23]; Oldroyd et al. [24]). Distinct trajectory subgroups have also been identified (Oldroyd et al. [24]). Prognostically, loss of independent ambulation occurs more quickly in men and in patients with older disease onset. Immunosuppression did not confer benefit and was associated with greater disability in a large observational cohort, although confounding by indication cannot be excluded (Benveniste et al. [25]). This cohort reported a median of approximately 14 years from onset to wheelchair use, with near-normal life expectancy in typical cases. Independent walking is often lost within about seven years (Cortese et al. [18]). Early-onset disease is more aggressive, with reduced survival (median age at death 59 years) despite otherwise similar pathology (Lindgren et al. [26]). Dunlap et al. [27] demonstrated a direct relationship between strength and function, showing that knee-extension strength declined as patients transitioned to more supportive gait devices (below approximately 30 N·m for a cane, 20 N·m for a walker) and that this decline was much faster than that of handgrip strength.

### 3.2. Motor Performance: Gait and Transitional Tasks (A2)

Motor-performance studies indicate that lower-limb function in IBM is primarily determined by quadriceps strength and the compensatory mechanisms it necessitates. Bernhardt et al. [28] demonstrated that as knee-extensor strength declines, sagittal knee mechanics progressively deteriorate, resulting in knee hyperextension during stance and reversal of the internal knee moment. Compensation occurs through increased reliance on hip and ankle musculature, with knee position at loading strongly correlated with hip-flexor and ankle-dorsiflexor strength (R ≈ 0.85). Gait is significantly slower than normal, and lower-extremity forces are correlated with temporal gait variables. Once the quadriceps are severely weak, ankle plantar-flexors and knee flexors are most strongly associated with walking performance (Davenport et al. [29]). Quadriceps strength predicts walking capacity, as evidenced by its correlation with 2 min and 6 min walk distances (r ≈ 0.58–0.60), and the quadriceps-to-hamstring ratio is inverted compared to normal (Lowes et al. [30]). Jørgensen et al. [31] identified the timed up-and-go (TUG) as the strongest predictor of self-reported physical function (accounting for 56% of variance), and knee-extensor strength asymmetry as the strongest predictor of walk distance. Conference-level data further support these patterns of lower-extremity weakness (Oh et al. [32]; Lowes et al. [33]).

### 3.3. Falls and Balance (A3)

Falls are nearly universal among individuals with IBM and are inadequately managed. In a national survey, 98% of respondents reported a history of falls, 60% experienced falls at least every three months, and only 18% had received any form of falls management (Hiscock et al. [34]). In a clinical cohort, 40% reported at least one fall within six months, with falls concentrated at intermediate IBM-FRS levels. Fear of falling was strongly correlated with the TUG (r = 0.90) and inversely correlated with IBM-FRS (r = −0.82) (Hunn et al. [35]). Fall-related injuries are substantial: a cross-sectional myopathy survey found that IBM carried a higher fall risk than dermatomyositis (odds ratio 2.5) and a 25.7% prevalence of fall-related fractures (Nandakumar et al. [36]). Another myopathy cohort identified sporadic IBM as having the most severe walking impairment and among the highest fall and fracture rates (median 2.5 falls per year; fractures in approximately 38%) (Danckworth et al. [37]). A single case report highlights that IBM frequently presents after a fall attributed to aging (Saiki et al. [38]). Fall ascertainment in these studies was predominantly retrospective and self-reported, with recall windows ranging from three to twelve months and non-uniform definitions of a fall; therefore, prevalence estimates should be interpreted as approximate. Measured postural balance or posturography in IBM was essentially absent from the eligible literature despite a dedicated search (arm A4), representing a significant evidence gap. In contrast, sensory and peripheral-nerve function have been measured directly and are synthesized separately under A4 (Section 3.4).

### 3.4. Lower-Limb Sensory, Proprioceptive, and Peripheral-Nerve Function (A4)

Two studies have directly assessed lower-limb sensory or peripheral-nerve function in sporadic IBM, both focusing on the feet and/or legs and thus aligning with the review’s lower-limb dysfunction scope. Arnardottir, Svanborg, and Borg [39] conducted sensibility screening and quantitative somatosensory-threshold testing in nine patients, finding abnormal sensibility in eight of nine (primarily thermal, in hands and/or feet), abnormal vibratory thresholds in five, and abnormal thermal thresholds in four. Mean vibratory thresholds were significantly higher than in controls (*p* < 0.05), with elevated heat-pain thresholds; the authors interpreted these findings as indicative of large-fiber peripheral-nerve involvement. Sturm et al. [40] performed a multimodal assessment of nineteen patients, including nerve-conduction studies, thigh and lower-leg skin biopsy, quantitative sensory testing, and corneal confocal microscopy. They identified large-fiber neuropathy in 67%, small-fiber pathology on skin biopsy in 72% (and in 32% by corneal confocal microscopy), and quantitative sensory testing indicated predominantly large-fiber damage (61%). Collectively, these studies provide direct evidence that both large- and small-fiber sensory involvement are common in IBM. However, both studies are small, single-center, and either uncontrolled or minimally controlled cross-sectional studies (certainty very low; Section 3.8). Importantly, neither study measured proprioceptive acuity (joint-position sense) or postural balance/posturography, which remain unassessed in IBM. Consequently, the relationship between sensory involvement and balance or falls is not established by direct evidence and is instead developed as a hypothesis in Section 4.2.

### 3.5. Intervention Effects (A5)

#### 3.5.1. Pharmacological Interventions

No pharmacological agent has demonstrated a convincing functional benefit in controlled trials. Randomized trials of intravenous immunoglobulin (IVIg) were neutral regarding primary strength outcomes (Dalakas MC et al. [41]; Walter et al. [42]), including when combined with prednisone (Dalakas et al. [43]). However, a functionally important subset showed transient improvement (Dalakas MC et al. [41]), and dysphagia occasionally responded. Results from uncontrolled series were mixed (Soueidan et al. [44]; Amato et al. [45]; Dobloug et al. [46]), and subcutaneous immunoglobulin produced small gains, with the largest effects observed in IBM among mixed myopathies (Cherin et al. [47]; Cherin et al. [48]). Bimagrumab increased muscle mass but not function: the phase 2b RESILIENT trial found no difference in 6 min walk distance compared to placebo (Hanna et al. [49]), and the long-term extension showed increased muscle mass without functional improvement (Sivakumar et al. [50]), despite an early single-dose signal (Amato et al. [51]). Arimoclomol did not slow IBM-FRS decline in a phase 2/3 trial (Machado et al. [52]). Alemtuzumab produced a transient reduction in strength decline that was not sustained (Dalakas et al. [53]). Sirolimus did not improve its primary outcome, maximal voluntary isometric knee-extension strength, in a phase 2b randomized, double-blind, placebo-controlled trial (*n* = 44), but several secondary measures favored sirolimus, including 6 min walk distance, forced vital capacity, HAQ-DI, and thigh fat fraction (Benveniste et al. [54]), consistent with a subsequent case report (Pawlitzki et al. [55]). A phase III trial (OPTIMISM) is ongoing (Badrising et al. [56]). Pioglitazone did not meet its primary molecular endpoint but showed pathway activation (Adler et al. [57]). Follistatin gene therapy improved 6 min walk distance in an open-label trial (Mendell et al. [58]). Testosterone produced no functional benefit (Connor et al. [59]), and ABC008 achieved its immunological target in a first-in-human study (Goel et al. [60]).

#### 3.5.2. Non-Pharmacological Interventions

Exercise interventions appear consistently safe in the studied populations, do not accelerate disease progression, and may help preserve function. Progressive resistance training improved strength in the least-affected muscles (Spector et al. [61]), and a home-based functional program improved both strength and mobility (Johnson et al. [62]). A pilot study found no significant change but also no deterioration (Arnardottir et al. [63]), and a case report suggested maintenance of strength (D’Alton et al. [64]). Blood-flow-restricted resistance training preserved knee-extensor strength, which declined in controls, and favored the IBM-FRS in an exploratory randomized trial (Jørgensen et al. [65]), consistent with case-level gains (Gualano et al. [66]; Santos et al. [67]). Aerobic training improved peak oxygen uptake with a strong effect size in the IBM group of a randomized feasibility trial (Wallace et al. [68]). Assistive strategies also show preliminary promise: stance-control orthoses address knee buckling (Bernhardt et al. [69]); robotic gait training with the Hybrid Assistive Limb improved ambulation and 2 min walk distance in a crossover trial (Nakajima et al. [70]) and a case series (Suzuki et al. [71]); and longer daily rehabilitation was associated with greater gains in activities of daily living in an administrative cohort (Tani et al. [72]). A small arimoclomol proof-of-concept trial (Machado et al. [73]) and a blood-flow-restriction pilot (Liang et al. [74]) were consistent in direction but small and exploratory.

### 3.6. Characteristics and Evidence Tables of Included Studies

Table 1 provides characteristics for all 64 included studies, grouped by the review question each principally informs and showing every question it contributes to. Studies are the unit of analysis: companion and duplicate reports were collapsed to their parent study, so the count of studies is smaller than the count of source reports. Because contributions overlap, several studies appear under one primary heading but are tagged for more than one question (Q column); across the set, 16 studies inform A1, 9 A2, 6 A3, 38 A5, and 2 A4.

The intervention studies (A5) are presented in three dedicated tables. Pharmacological trials are summarized separately from rehabilitation studies in Table 2a. Exercise and training studies are detailed in a dedicated intervention table that reports the frequency, intensity, time, type, progression, supervision, comparator, adherence, safety, and outcomes of each program (Table 2b, landscape). Assistive-device and robotic studies are included in Table 2c. Where available, we report the number analyzed, the exact follow-up period, and the effect estimate with its 95% confidence interval. This approach allows for clear differentiation between precise controlled estimates and uncontrolled case-level observations or exploratory conference abstracts. Studies that report only within-group change, lack a comparator, or do not provide a measure of uncertainty are marked accordingly (single-arm, *n* = 1, or NR).

### 3.7. Risk of Bias

Studies were appraised at the level of the specific outcome relevant to each review question, employing the instrument appropriate for each study design (Table 3). RoB 2 was used for randomized trials, ROBINS-I for non-randomized intervention studies, QUIPS for prognostic or natural-history cohorts, and the corresponding JBI critical-appraisal checklists for analytical cross-sectional studies and for case series or reports. Judgments were made at the domain level and accompanied by written justifications. Full item-level justifications are available in the risk-of-bias workbook accompanying this review. Numerical scoring and percentage thresholds were not applied. Conference abstracts were not formally appraised due to incomplete reporting of methods; these were flagged and did not carry the same evidential weight as full reports.

Across the 56 full reports, methodological quality was modest and varied by design. Of the 13 randomized trials appraised with RoB 2, three were at low risk of bias, eight raised some concerns, and two were at high risk; the concerns arose chiefly from small samples, incompletely reported randomization and allocation concealment, and exploratory, non-prespecified efficacy endpoints. The 12 non-randomized intervention studies (ROBINS-I) were uniformly weak—eleven at serious and one at critical risk of bias—reflecting uncontrolled designs, confounding by indication (particularly for corticosteroids and immunoglobulin), and single-arm follow-up. Among the 10 prognostic and natural-history cohorts (QUIPS), five were at moderate and five at high risk, limited by attrition, single-center recruitment, and unadjusted confounding. The 11 analytical cross-sectional studies (JBI) were at moderate (*n* = 7) or high (*n* = 4) risk, driven by small convenience samples and unadjusted correlations, and all 10 case series or case reports were at high risk given their inherent design. The eight conference abstracts were retained as clearly labeled gray literature and were not formally appraised, because their methods were too incompletely reported to support a confident judgment; they were not given the same evidential weight as full reports. Overall, no body of evidence rested on a foundation of consistently low risk of bias, and the strongest designs—randomized trials of pharmacological agents—were concentrated in the intervention question (A5).

### 3.8. Certainty of Evidence (GRADE)

The GRADE framework was adapted to rate certainty separately for intervention questions and for descriptive or prognostic questions, without transferring certainty ratings between these categories (Table 4). For intervention outcomes (A5), evidence from randomized trials was initially rated as high certainty, while non-randomized (uncontrolled) evidence began at low certainty. Certainty was subsequently rated down for risk of bias, inconsistency, indirectness, imprecision, and publication bias; no domain was rated up. Specifically, no observational body was upgraded for a “large effect” because no pooled estimate or prespecified large-effect threshold was defined, and the included studies employed heterogeneous designs and outcomes. Patient-important benefits and harms were assessed independently. For prognostic or natural-history outcomes (A1) and falls burden (A3), the GRADE framework for overall prognosis and prognostic-factor evidence was applied, with cohort evidence beginning at high or moderate certainty and rated down as appropriate. These ratings describe the certainty in the *estimated rate or association*, rather than a treatment effect. When no eligible IBM-specific study was identified for a concept included in the search (such as measured postural balance or posturography in arm A3, or task-specific/transfer rehabilitation and dedicated balance training in arm A5a), this was reported as a searched-but-empty evidence gap, rather than as a low-certainty estimate of effect. Proprioceptive, sensory, and neurological function were searched directly in IBM (Appendix A, arm A4); direct sensory or peripheral-nerve evidence is synthesized as question A4 (Section 3.4), while proprioceptive acuity and posturography remain unmeasured and represent residual evidence gaps. Diagnostic phenotype, biomechanical mechanism, and non-IBM or animal material were not included in any GRADE profile.

Certainty of evidence was low or very low for most outcomes, and no outcome reached high certainty. The highest ratings—moderate—were attached not to demonstrated benefits but to well-conducted trials showing an absence of functional benefit: bimagrumab did not improve the 6 min walk distance, and arimoclomol did not slow IBM-FRS decline (both moderate certainty, including for their adverse-event profiles). Intravenous immunoglobulin, sirolimus (maximal knee-extension strength), and testosterone were supported by low-certainty evidence, whereas alemtuzumab, follistatin gene therapy, pioglitazone, ABC008, and the secondary walk-distance signal for sirolimus were very low. For non-pharmacological interventions, aerobic training (aerobic capacity), blood-flow-restricted resistance training (strength), and the safety of conventional resistance exercise were rated low, while the muscle-strength effect of conventional resistance exercise, the harms of blood-flow-restricted training, stance-control orthoses, and robotic (HAL) gait training were very low. Among descriptive questions, the rate of quadriceps strength decline in natural-history cohorts reached moderate certainty—the single most robust finding in the review—whereas IBM-FRS-based functional decline (A1), the association of lower-limb strength with mobility (A2), and the prevalence and burden of falls (A3) were low, and directly measured lower-limb sensory and peripheral-nerve function (A4) was very low. Concepts that were searched but returned no eligible IBM study—objective postural balance/posturography, proprioceptive acuity, and task-specific or dedicated balance-training and falls-prevention interventions—were recorded as evidence gaps rather than assigned a certainty rating. In sum, the most secure conclusions are that the pharmacological agents tested to date do not meaningfully improve lower-limb function and that quadriceps strength decline is the most reliably quantified marker of progression, while evidence on rehabilitation, balance, falls, and sensory contributions remains low to very low.

## 4. Discussion

### 4.1. Principal Findings

Analysis of 64 IBM-specific studies shows four consistent findings. First, quantitative quadriceps strength and validated functional scales such as the IBM-FRS are the most sensitive markers of disease progression and are frequently utilized to inform prognostic counseling and clinical trial design. Second, gait disturbances, difficulties with transfers, and frequent falls are commonly observed in the context of quadriceps failure; knee-extensor weakness is closely linked to knee instability and increased reliance on gait aids, while falls are prevalent and often insufficiently addressed. Third, the intervention literature is characterized by small, uncontrolled studies and neutral randomized trials; no pharmacological agent has shown a clear functional benefit, whereas exercise interventions (including blood-flow-restricted and aerobic training), orthoses, and robotic assistance have been reported as safe and may help maintain mobility. Fourth, direct lower-limb testing indicates that both large- and small-fiber sensory and peripheral-nerve dysfunction are common in IBM, yet proprioceptive acuity and postural balance have not been systematically measured; therefore, the sensory contribution to instability is established at the nerve level but remains untested at the functional level.

### 4.2. A Conceptual Model of Mobility Loss and Falls in IBM

**Figure 2:**
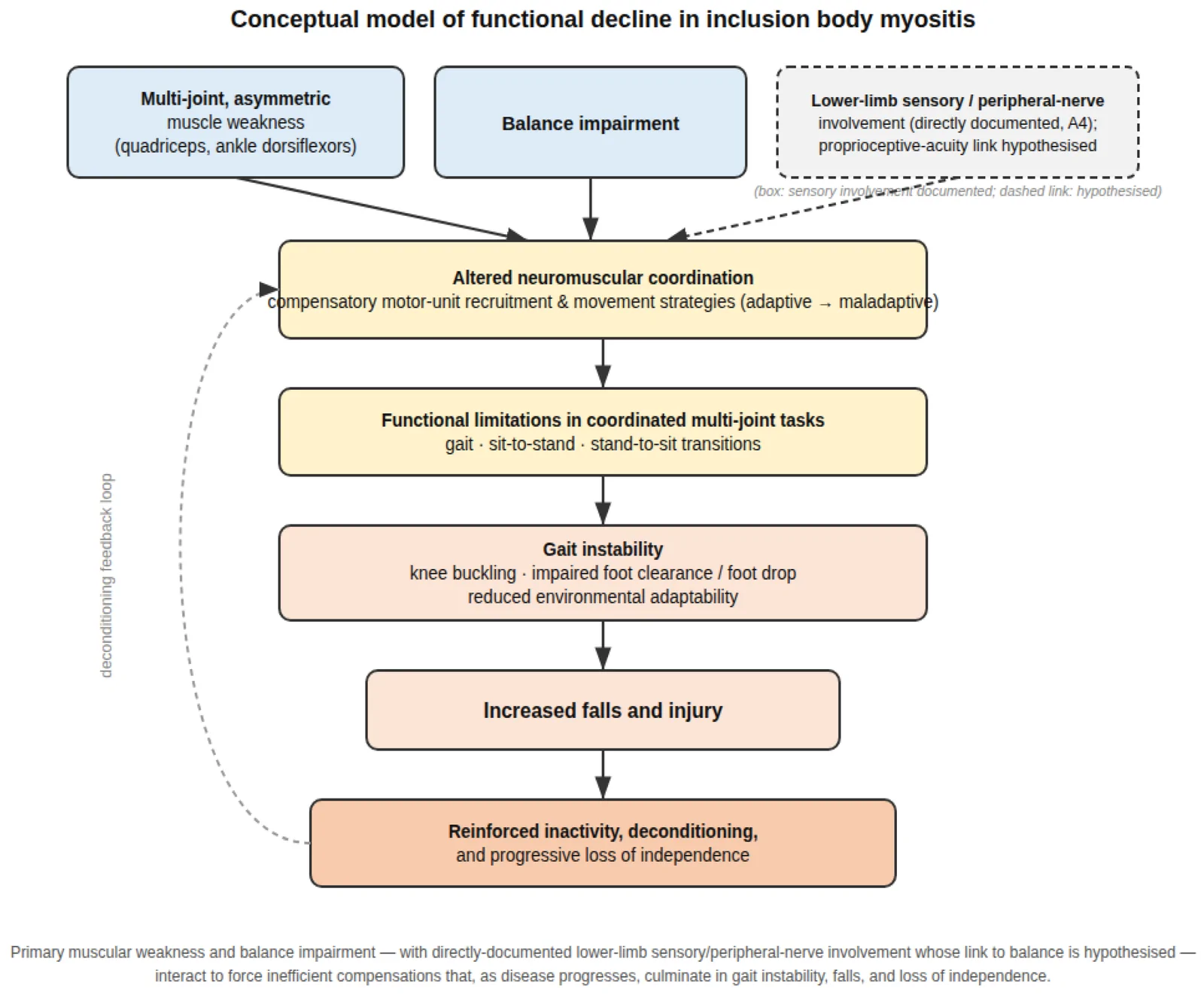
Hypothesized conceptual model of the possible progression of mobility and independence loss in inclusion body myositis (IBM). Three contributing factors are shown at the top: multi-joint, asymmetric muscle weakness (quadriceps and ankle dorsiflexors) and balance impairment—both directly documented in the synthesized evidence (solid boxes)—together with lower-limb sensory and peripheral-nerve involvement (dashed box), for which the sensory abnormalities are documented but the proposed link to balance is hypothesized (dashed arrow). These factors are depicted as converging on altered neuromuscular coordination—a proposed shift in compensatory motor-unit recruitment and movement strategies from adaptive toward maladaptive—which is, in turn, linked, in sequence, to functional limitations in coordinated multi-joint tasks (gait, sit-to-stand, and stand-to-sit), gait instability (knee buckling, impaired foot clearance or foot drop, and reduced environmental adaptability), increased falls and injury, and ultimately reinforced inactivity, deconditioning, and progressive loss of independence. A dashed feedback loop denotes a proposed deconditioning cycle returning to altered neuromuscular coordination. All relationships are offered for hypothesis generation only and do not represent demonstrated causal pathways.

The objective findings—decline in quadriceps strength, its biomechanical impact on gait and transfers, and the associated increase in falls—are organized within a conceptual model describing the possible progression of independence loss in IBM (see Figure 2). In this framework, functional decline is hypothesized to be associated with the interaction of multi-joint, asymmetric muscle weakness, balance impairment, and a potential proprioceptive or sensory contribution, rather than just isolated muscle weakness. These primary impairments are proposed to be associated with compensatory changes in motor-unit recruitment and movement strategies, which may initially be adaptive but potentially become maladaptive as the disease advances. Such compensations may present as deficits in coordinated multi-joint tasks (e.g., gait, sit-to-stand, and stand-to-sit), progressing to gait instability (manifested by knee buckling, impaired foot clearance, foot drop, and reduced adaptability to environmental obstacles), and ultimately to falls and injury, which may be linked with inactivity, deconditioning, and loss of independence. The muscular and balance aspects of this model are supported by the direct evidence synthesized in this review; the proprioceptive component is included as a hypothesized, secondary pathway, as it has not yet been directly demonstrated in IBM.

The following material is excluded from the primary evidence base and is presented as mechanistic context and hypothesis, with individual citations, in accordance with the scope of this review.

The proprioceptive/sensory hypothesis and its direct IBM support. It has been proposed that proprioceptive and neurological change may compound weakness in destabilizing the knee and provoking falls in IBM. The direct evidence synthesized under A4 (Section 3.4) shows that lower-limb sensory and peripheral-nerve function have been measured in IBM and are frequently abnormal: Arnardottir, Svanborg and Borg [39] found abnormal quantitative somatosensory thresholds—raised vibratory thresholds in five of nine patients and elevated heat-pain thresholds—concluding a large-fiber peripheral-nerve affection; and Sturm et al. [40], combining nerve-conduction studies, thigh and lower-leg skin biopsy, quantitative sensory testing and corneal confocal microscopy, identified large-fiber neuropathy in 67% and small-fiber skin-biopsy pathology in 72% of IBM patients. What remains untested is the link from such sensory involvement to proprioceptive acuity, postural control, and falls: no IBM study has measured joint-position sense, motion-detection thresholds, or posturographic sway. Evidence for that link is still indirect and drawn from other conditions with selective muscle loss, which differ from IBM and are cited here only to motivate hypotheses. Butler et al. [80] showed that individuals with post-polio syndrome and severe ankle-dorsiflexion weakness had increased postural sway with eyes closed, implicating proprioceptive instead of purely strength-related deficits; Hassan et al. [81] found that patients with knee osteoarthritis had reduced quadriceps strength, impaired proprioceptive acuity, and increased sway relative to controls; and studies of chronic ankle instability link unilateral weakness to measurable proprioceptive deficits on the affected side (Liu et al. [82]; Santos & Liu [83]; Sousa et al. [84]). Plantar-sensation loss has similarly been associated with gait instability in older adults (Franz et al. [85]). None of these establishes causality or applies directly to IBM; at most, they suggest that selective muscle loss may coexist with impaired proprioception and postural control. Because direct data are lacking, three testable hypotheses are proposed for future study rather than presented as findings: (1) IBM patients may have reduced proprioceptive acuity (joint-position sense, motion-detection thresholds) compared to age-matched controls; (2) proprioceptive impairment may be associated with the severity of quadriceps and ankle-dorsiflexor weakness; and (3) strength asymmetry may be associated with asymmetry in proprioceptive accuracy and postural sway.

Neurogenic features. A possible primary neurogenic component in IBM remains debated. Needle electromyography has identified “pseudo-neurogenic” motor-unit potentials of longer duration and stage-dependent change (Soltanzadeh et al. [86]; Nojszewska et al. [87]; Mano et al. [88]), and imaging and neurophysiological studies describe lower-limb nerve hypertrophy and features of an accompanying polyneuropathy or large-fiber nerve involvement (Elmansy et al. [89]; Abualhayjaa et al. [90]; Lee et al. [91]). Where large-fiber sensory loss occurs, it can produce a broad-based, slow, “stomping” gait resembling advanced IBM, making it plausible—but unproven—that any sensory contribution would degrade joint-position detection, foot-placement timing, and rapid postural adjustment. These electrodiagnostic studies are cross-sectional and do not measure balance or falls; however, taken with the direct sensory studies above (Arnardottir, Svanborg & Borg [39]; Sturm et al. [40]), they establish that measurable large- and small-fiber abnormality is common in IBM, while leaving the link to postural control untested. Not every neurological correlate is relevant: an immunological study of muscle dendritic-cell populations, for example, characterizes disease activity rather than sensory function (Kirou et al. [92]). A recent systematic review reached a concordant conclusion—that quadriceps and ankle weakness impair balance and raise fall risk in IBM while rehabilitation evidence remains limited (Jeon et al. [6]).

Task-specific motor coordination and compensation are important in gait and transitional movements, which call for precise, task-specific activation patterns—such as swing-phase hip flexion followed by controlled knee extension and dorsiflexor-mediated foot clearance during gait, and coordinated knee, hip, and trunk extension during sit-to-stand. When selective weakness or sensory changes occur, patients are observed to adopt compensatory strategies. According to established motor-control frameworks, these compensations may be adaptive (maintaining task performance without instability) in early stages, but potentially maladaptive (increasing joint stress, asymmetry, and fall risk) as the disease progresses. Quadriceps weakness is associated with impaired controlled knee extension during weight acceptance, greater reliance on hip extensors and ankle dorsiflexors, and an elevated risk of knee buckling. Concurrent dorsiflexor weakness is associated with impaired toe clearance and difficulties with controlled foot lowering. The hypothesized transition from adaptive to maladaptive compensation is presented as a rationale—based on identified biomechanical failure modes rather than proven efficacy—for task-specific, transfer-focused rehabilitation and the use of external supports such as orthoses and robotic assistance, consistent with the low-certainty findings in the intervention literature.

The deficits observed in IBM mirror, but progress more rapidly than, those seen in normal aging. Jensen et al. [93] found that IBM patients exhibit gait speed, chair-stand performance, and leg power comparable to individuals approximately fifteen years older, highlighting the phenomenon of accelerated functional aging. Population data, including a high mitochondrial-DNA mutation burden and reduced survival in early-onset cases (Lindgren et al. [26]), further support the presence of a degenerative process occurring alongside inflammation. In non-IBM older adults, assistive-device use is associated with a more conservative, slower walking pattern, as well as increased falls and fear of falling (Liu et al. [94]; Roman de Mettelinge & Cambier [95]), paralleling the earlier and more rapid device dependence observed in IBM. This dual-pathology model, encompassing both inflammatory and degenerative components, helps to explain the central paradox in the intervention literature: while immunotherapies targeting inflammation have consistently failed to modify the functional trajectory, anabolic and rehabilitative strategies designed for muscle preservation and use have produced more promising, though still preliminary, results.

### 4.3. Task-Specific Coordination and the Adaptive-to-Maladaptive Compensation Model

**Figure 3:**
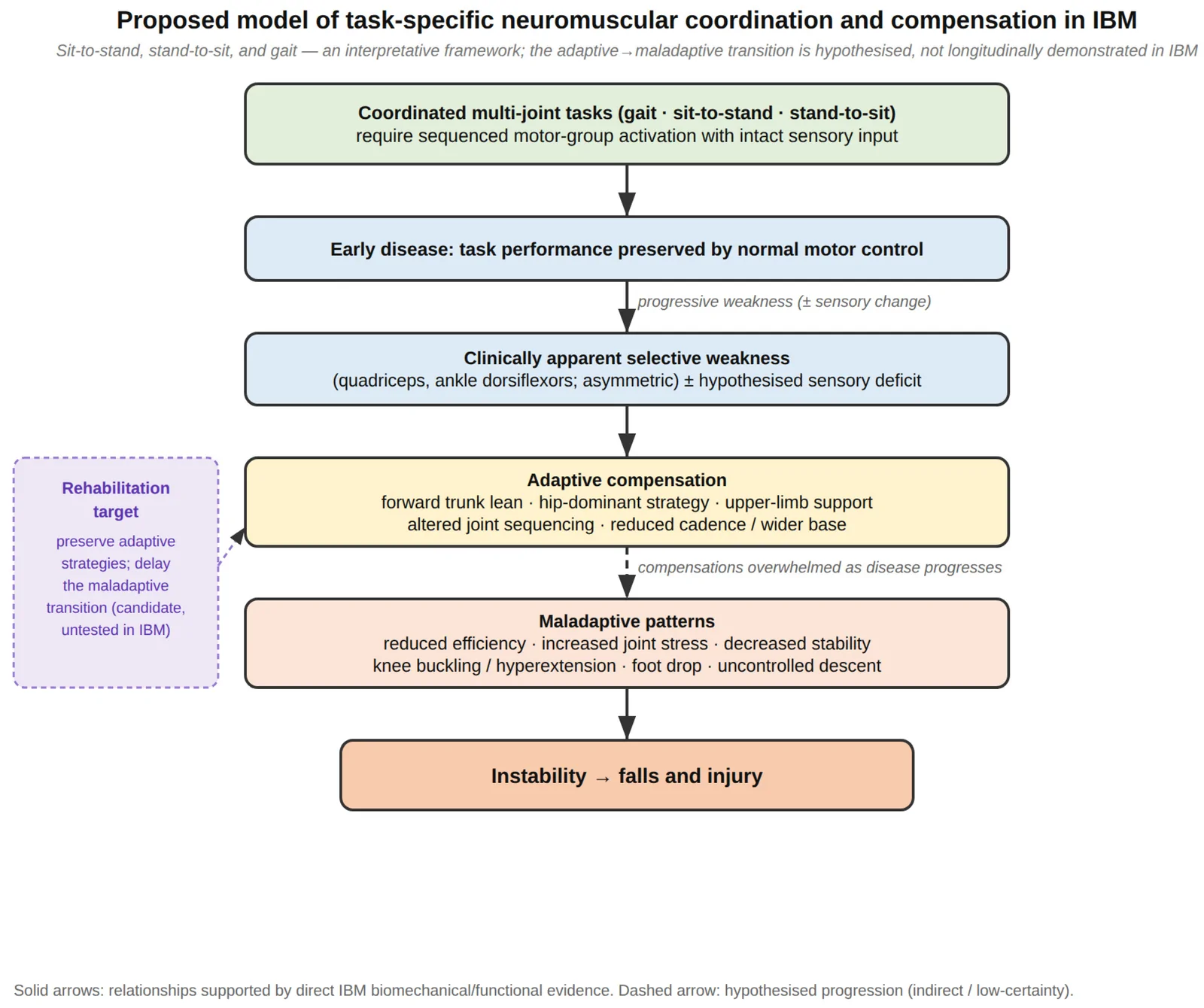
Proposed interpretative framework for the hypothesized transition from adaptive to maladaptive compensation in task-specific motor coordination in IBM. Read from top to bottom, coordinated multi-joint tasks (gait, sit-to-stand, and stand-to-sit) are shown to require sequenced motor-group activation with intact sensory input; in early disease, task performance is depicted as preserved by normal motor control, which—with progressive weakness and possible sensory change—is proposed to give way to clinically apparent selective weakness (quadriceps and ankle dorsiflexors, asymmetric, with a hypothesized sensory deficit). This is proposed to prompt adaptive compensation (forward trunk lean, hip-dominant strategy, upper-limb support, altered joint sequencing, and reduced cadence or a wider base), which as disease progresses may be overwhelmed and give way to maladaptive patterns (reduced efficiency, increased joint stress, decreased stability, knee buckling or hyperextension, foot drop, and uncontrolled descent) and thereby to instability, falls, and injury. A side panel identifies adaptive compensation as a candidate rehabilitation target—preserving adaptive strategies to delay the maladaptive transition—an approach that remains untested in IBM. Solid arrows denote relationships supported by direct IBM biomechanical or functional evidence; the dashed arrow denotes the hypothesized progression, which rests on indirect, low-certainty evidence rather than an established causal relationship.

Gait, sit-to-stand, and stand-to-sit are highly coordinated multi-joint tasks that depend on correctly sequenced activation of hip, knee, and ankle musculature supported by intact sensory input. The descriptions of normal task biomechanics below are general movement science, not evidence derived from the included IBM studies; they are used here only to interpret the IBM-specific findings synthesized in the Results Section. Within that frame, the review’s direct findings—decreased gait speed and increased gait-cycle time, the association of ankle plantar-flexor strength with walking speed and knee-flexor strength with temporal parameters (Davenport et al. [29]), and the association of knee-extension strength and limb asymmetry with impaired timed-up-and-go and sit-to-stand performance (Jørgensen et al. [31]; Lowes et al. [30])—can be organized into a proposed model of how coordinated performance is lost (Figure 3).

In this model, early task performance is described as being maintained through compensatory mechanisms such as forward trunk lean, hip-dominant strategies, upper-limb support, and altered joint sequencing. As selective weakness (and any sensory changes) progresses, these compensations may become increasingly stressed and mechanically inefficient, which may be associated with gait instability (knee buckling and hyperextension, foot drop and impaired clearance, and uncontrolled descent) and, ultimately, falls. Several cautions are essential. First, whether a given strategy is adaptive or maladaptive is context-dependent: knee hyperextension increases joint stress but can prevent collapse; trunk lean reduces knee-extensor demand but raises energy cost; upper-limb support preserves transfer independence despite distal weakness. A strategy should not be considered maladaptive solely because it is asymmetric or mechanically inefficient—in progressive disease, an asymmetric strategy may represent the safest available adaptation. Second, the assumption of preserved “normal motor control” in early disease is not directly demonstrated; subclinical adaptation may precede clinically apparent weakness. Third, and most importantly, the available evidence is cross-sectional: it supports the interpretation that disease progression is associated with both greater weakness and greater reliance on compensation but does not demonstrate that compensation itself causes decline. Compensation may therefore be a marker or consequence of progression rather than its cause, and no causal or temporal claim can be made without longitudinal, mediation-capable data. Accordingly, Figure 3 is presented as a proposed interpretative framework derived from indirect and low-certainty evidence, with solid arrows indicating relationships supported by direct IBM biomechanical/functional data and the dashed arrow representing the hypothesized adaptive-to-maladaptive transition. Real-world studies tracking the evolution of these strategies across operationally defined disease stages, on variable terrain and during obstacle negotiation, are lacking and are a priority for future work.

### 4.4. Candidate Rehabilitation Strategies: A Research Agenda

**Figure 4:**
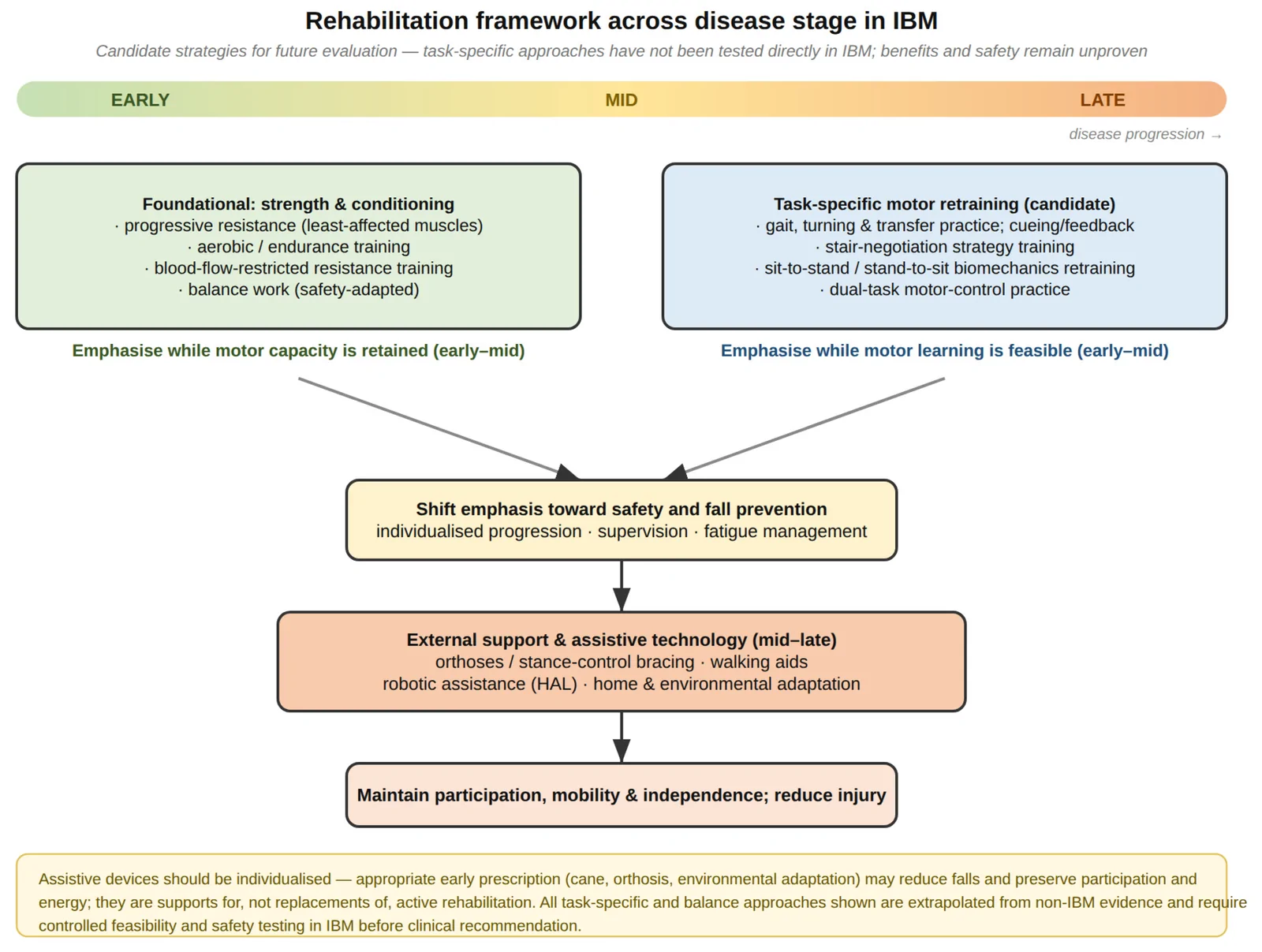
Framework organizing candidate rehabilitation strategies in IBM as hypothesized targets for future evaluation, arranged against disease stage (early to late). While motor capacity and motor learning are retained (early-to-mid stage), two emphases are proposed in parallel: foundational strength and conditioning (progressive resistance in least-affected muscles, aerobic and endurance training, blood-flow-restricted resistance training, and safety-adapted balance work) and candidate task-specific motor retraining (gait, turning, and transfer practice with cueing or feedback; stair-negotiation strategy training; sit-to-stand and stand-to-sit biomechanics retraining; and dual-task motor-control practice). As disease progresses, the framework depicts a shift of emphasis toward safety and fall prevention (individualized progression, supervision, and fatigue management), and then toward external support and assistive technology in the mid-to-late stage (orthoses and stance-control bracing, walking aids, robotic assistance such as the Hybrid Assistive Limb, and home and environmental adaptation), with the overall aim of maintaining participation, mobility, and independence while reducing injury. Assistive devices are framed as individualized supports for—not replacements of—active rehabilitation. All task-specific and balance approaches shown are extrapolated from non-IBM evidence and would require controlled feasibility and safety testing in IBM before clinical recommendation; none represents a demonstrated treatment effect.

The intervention evidence synthesized in the Results Section supports two conclusions with reasonable confidence: no pharmacological agent has convincingly altered the functional trajectory, and conventional exercise (including blood-flow-restricted and aerobic training) is safe in the studied populations and may help preserve strength and capacity. Beyond this, a range of task-specific and balance-oriented strategies has been proposed but not evaluated directly in IBM; the framework in Figure 4 organizes them as candidate interventions for future evaluation, not as current recommendations, and every element requires controlled feasibility and safety testing before clinical use. It is worth stating plainly which of these rest on IBM evidence and which do not: conventional resistance, aerobic, and blood-flow-restricted training have at least been tested in IBM (in small, mostly uncontrolled studies of low certainty), whereas task-specific retraining, stair and dual-task training, and dedicated balance training have not been evaluated in IBM at all and are plausible-but-untested candidates whose volume in the literature should not be mistaken for an existing evidence base.

Two cautions from the direct evidence should temper enthusiasm. First, much of the conventional-exercise signal comes from small, uncontrolled, within-group studies; within-group improvement is not a between-group treatment effect, and natural fluctuation, learning effects, concurrent therapy, selection, and regression to the mean are all plausible alternative explanations. Where safety was reported, exercise (including blood-flow-restricted protocols) was generally well tolerated, but adverse events should be synthesized from the actual included studies rather than from general precautionary statements or from risks extrapolated from other populations. Second, several commonly proposed balance and task-specific techniques carry population-specific safety concerns: single-leg stance, unstable-surface work, reduced-visual-input drills, and perturbation training may be unsafe in patients with marked quadriceps weakness; dual-task practice may increase fall risk and should be tested in a controlled feasibility study first; and training toward symmetric weight distribution may not be optimal for a patient whose safest strategy is asymmetric. Task-specific motor retraining, stair-negotiation strategy training, and sit-to-stand/stand-to-sit biomechanics retraining are biologically reasonable candidates precisely because they target the coordination failures identified above, but their rationale is currently extrapolated from the larger neuromuscular and motor-learning literature. Assistive devices deserve careful, individualized framing: rather than being “reserved” until capacity has declined, an individualized and appropriately timed device (cane, orthosis, stance-control bracing, or environmental modification) may reduce falls, preserve participation, conserve energy, and prolong community mobility, and should be seen as a support for—not a replacement of—active rehabilitation; any disuse effect is more likely to be associated with disease progression than with the device itself.

Indirect evidence from other neurological and musculoskeletal populations can help frame—but cannot establish—this agenda and is cited here solely as rationale. Structured, functionally stratified group exercise has been shown to be feasible and to improve strength and endurance in hospitalized spinal-cord-injury patients (Garay-Sánchez et al. [96]), supporting the case for stratified rehabilitation pathways that distinguish ambulatory from non-ambulatory IBM patients; task-specific gait training evaluated through clinically meaningful mobility outcomes in spinal cord injury (Echemendía del Valle et al. [97]) illustrates how walking, transfer, and repeated-task practice might be assessed in future IBM trials; and the coexistence of impaired lower-limb performance with altered spinal alignment and whole-body postural organization in Parkinson’s disease (Bissolotti et al. [98]) supports a nuanced view in which functional decline reflects interactions among lower-limb capacity, trunk position, and postural control rather than the strength of a single muscle group. None of these were conducted for IBM, and improvements observed in another condition cannot be assumed to occur in a progressive myopathy. The priority, therefore, is a small set of well-designed feasibility and efficacy trials that test these stage-adapted, task-specific strategies directly in IBM, with function, falls, participation, and safety as endpoints.

### 4.5. The Failing-Compensation Model in Context

The interpretation presented here—that independence may be lost through the progressive failure of compensatory mechanisms necessitated by selective weakness—is not intended to replace the accepted understanding of IBM as a primary myopathy. Progressive muscle degeneration with inflammation is considered the root cause of the disease and is understood to underlie both the weakness and the compensatory strategies observed. The compensation model is presented as a conceptual framework for understanding how primary muscle loss could be associated with functional decline and falls, rather than as a competing definition of the disease. The two perspectives generate partially separable predictions, and the data required to distinguish between them are longitudinal and mediation-capable: if failing compensation is associated with decline beyond weakness itself, measures of movement efficiency, asymmetry, and postural control would be expected to predict falls and mobility loss after adjustment for quadriceps strength, and interventions that improve coordination without changing strength would be expected to improve function. No IBM dataset currently permits this test, which is why the model is offered as a hypothesis for future study rather than as an established mechanism.

Placing the findings in the wider neuromuscular field sharpens both their novelty and their limits. IBM’s functional profile most closely resembles an accelerated form of age-related sarcopenia: quadriceps-predominant weakness, loss of muscle power, slowed gait, impaired chair-rise, and a high fall burden are characteristic of both conditions. IBM patients have been reported to perform at the level of individuals approximately fifteen years older (Jensen et al. [93]). This comparison is practically useful, as the sarcopenia field has validated outcome measures (gait speed, chair-stand time, leg power, and the Short Physical Performance Battery) and intervention strategies that the IBM evidence base still lacks, providing a template for endpoint selection and trial design. Nonetheless, IBM differs from sarcopenia in its selective, asymmetric distribution, its combined degenerative and inflammatory pathology, and its relative resistance to exercise interventions that benefit healthy older adults. As such, sarcopenia protocols cannot be applied without critical evaluation. When considered alongside other slowly progressive myopathies—including limb-girdle, oculopharyngeal, and distal myopathies—IBM is further distinguished by its early quadriceps and finger-flexor involvement and the high incidence of falls associated with knee-extensor weakness. However, it shares with these conditions the challenge of synthesizing evidence from small, single-center cohorts and heterogeneous, non-standardized outcomes. This review contributes by assembling, for IBM, the direct lower-limb functional evidence that is otherwise dispersed across more extensive and disease-adjacent studies.

### 4.6. Studies Excluded from the Direct-Evidence Base

Consistent with the scope, several IBM reports were assessed but not counted as review evidence and are instead cited above as mechanistic context. These include the electrodiagnostic, peripheral-nerve, and immunological studies (Soltanzadeh et al. [86]; Nojszewska et al. [87]; Mano et al. [88]; Elmansy et al. [89]; Abualhayjaa et al. [90]; Lee et al. [91]; Kirou et al. [92]); measurement-validation work; biomarker/diagnostic-only reports; and studies whose only outcomes were distal (finger-flexor/grip) or bulbar (dysphagia). Retaining these individually in the Section 4, rather than in the evidence tables, keeps the counted evidence focused on lower-limb function, mobility, balance, and falls while preserving the mechanistic narrative that gives those outcomes their biological meaning.

### 4.7. Strengths and Limitations

Strengths of this review include a focused synthesis of direct human IBM evidence, with non-IBM, animal, and secondary (narrative/systematic-review) sources kept outside the counted evidence base and used only as clearly labeled mechanistic context; study-level de-duplication, with companion reports collapsed so that trials and cohorts are not double-counted; a broad search across eight databases and two trial registries without outcome-word filters or a date limit; domain-level risk-of-bias judgments made with the design-appropriate instrument; GRADE profiles that report populations, comparators, participant numbers, and effect estimates with independent benefit and harm rows; a scope restricted to lower-limb function, mobility, balance, and falls; and explicit separation of direct evidence from hypothesis.

Important limitations remain and temper the conclusions. First, the review was not prospectively registered and no public dated protocol exists; although eligibility, mixed-cohort handling, appraisal, and certainty rules are reported in full, the absence of registration increases the possibility of post hoc decisions and, with it, the risk of selective emphasis—a risk that is heightened when synthesis is organized around a conceptual model, and which the SWiM-based, effect-oriented synthesis mitigates but cannot eliminate. Specifically, the review’s scope and structure were revised after the initial searches: the five review-question framework—including the lower-limb sensory and peripheral-nerve arm (A4)—was defined post hoc, and studies counted in the previous version were reclassified post hoc, with non-IBM, mechanistic, and secondary reports moved from the counted evidence base to clearly labeled Discussion-only context (documented study-by-study in Supplementary File S6); these decisions were made to sharpen the review’s identity and reduce double counting, but were not prespecified. Second, the evidence itself is weak: most studies are small, single-center, and uncontrolled; outcome measures and reporting are heterogeneous (precluding meta-analysis); and the natural-history literature is dominated by a few specialist centers. Third, diagnostic heterogeneity across successive (Griggs and ENMC) criteria limits comparability between older and newer studies. Fourth, despite study-level de-duplication, some participant overlap across publications from the same programs cannot be fully excluded, so unique-participant totals are approximate. Fifth, conference abstracts were retained as tagged gray literature but carry limited methodological detail and were not formally appraised, so their contribution is necessarily uncertain. Sixth, IBM-specific balance/posturography data are essentially absent despite being searched (a searched-but-empty gap); the sensory arm A4 found direct evidence of large- and small-fiber sensory/peripheral-nerve dysfunction in IBM, synthesized as a distinct strand (A4; Section 3.4), but the specific link from said sensory change to proprioceptive acuity, postural control and falls remains untested, and proprioceptive acuity and posturography themselves remain unmeasured. Seventh, the rehabilitation approaches discussed as candidates—task-specific retraining, stair and transfer training, dual-task practice, and dedicated balance training—are largely derived from non-IBM populations and have not been evaluated for feasibility or safety in IBM; they are framed here as a research agenda, not as recommendations. Finally, several patient-important dimensions lie outside the synthesized outcomes—fatigue, pain, dysphagia, fear of falling, participation and quality of life, caregiver burden, and the home environment—and screening, extraction, and appraisal, though performed in duplicate and reconciled by consensus between the two reviewers, were carried out by a single team rather than replicated independently.

### 4.8. Implications

For clinicians, quantitative quadriceps strength, the IBM-FRS, timed walk tests, and the TUG are the most informative measures of progression and function, and falls risk is high and actively manageable yet frequently unaddressed. Where the direct evidence supports a practical starting point, it is for moderate-intensity progressive resistance and aerobic training, typically two to three supervised sessions per week over 12–16 weeks—the range used in the IBM exercise and blood-flow-restriction studies (Johnson et al. [62]; Jørgensen et al. [65]; Wallace et al. [68])—with sub-maximal loads, the least-affected muscles prioritized, creatine kinase and fatigue monitored for overwork, and falls-prevention and appropriately-timed assistive devices integrated; blood-flow-restricted training can deliver a strength stimulus at lower mechanical load for patients who cannot tolerate heavy resistance. Task-specific, stair, dual-task, and balance approaches carry no IBM-specific dosing evidence and should be evaluated in supervised feasibility studies before routine clinical use. For researchers, the priorities are adequately powered trials of anabolic and rehabilitative interventions with function and falls as endpoints, standardized outcome reporting (borrowing validated measures from the sarcopenia field), and primary studies quantifying balance and proprioception to test—rather than assume—the sensory contribution to mobility loss.

## 5. Conclusions

IBM progression is most sensitively assessed using quantitative quadriceps strength and the IBM-FRS. Gait, transfers, and falls are frequently reported in the context of quadriceps failure. Despite numerous trials, no pharmacological agent has been shown to convincingly improve function, whereas exercise, orthotic, and robotic strategies are reported as safe and may help preserve function. A mechanistic model containing muscular, degenerative, and possible sensory contributions is used to contextualize these findings. The primary myopathy—progressive muscle degeneration with inflammation—is considered the principal underlying process, while failing compensation and sensory changes are described as potential consequences that may worsen muscle loss, rather than as primary causes. Several evidence gaps define the priorities for future research. Objective postural balance (posturography) and proprioceptive acuity (joint-position sense) have not been directly measured in IBM, so the relationship between sensory involvement, balance, and falls remains to be established. No dedicated balance-training, task-specific rehabilitation, or falls-prevention interventions have been tested in IBM despite falls being near-universal, and the available exercise, orthotic, and robotic evidence rests on small, uncontrolled studies. Adequately powered controlled trials of these strategies, together with longitudinal, mediation-capable studies, are needed to test the proposed model and to determine whether targeting compensation and balance can help preserve mobility and reduce falls.

## Figures and Tables

**Table 1:** Characteristics of all included studies, by review question.

**A1. Natural History & Progression**					
**Study**	**Design**	**IBM *n***	**Q(s)**	**Follow-Up**	**Principal Finding & Key Estimate (95% CI/SD/*p*)**
Allenbach (2012) [16]	Prospective cohort	22	A1	9 mo (2 visits)	Asymmetrical generalized weakness (whole composite 43.3 ± 16.5% predicted); knee-extension QMT −12.79 ± 14.80% over 9 mo (*p* = 0.026); whole-body strength −1.6%/9 mo; dominant side stronger.
Lilleker (2019) [23]	Retrospective cohort (abstract)	80	A1	Median 2.3 yr (IQR 0.6–4.4); 232 person-yr	Yearly strength decline greatest for pinch −16.0% (95% CI −18.4 to −13.5), grip −5.5% (−6.3 to −4.6), knee extension −7.1%.
Oldroyd (2020) [24]	Retrospective cohort	75	A1	Median 4 yr (IQR 3–6); 348 person-yr	Yearly decline: pinch −10.1% (95% CI −11.0 to −9.1), knee extension −4.2% (−5.0 to −3.4); elbow flexion and wrist extension spared.
Lowes (2012) [30]	Cross-sectional	85	A1, A2	Single session	Quadriceps strength correlated with 2MWT r = 0.603 and 6MWT r = 0.578 (both *p* < 0.001); mean quadriceps 18% predicted (range 0–85%); 93% < 50% predicted.
Jørgensen (2017) [31]	Cross-sectional	22	A1, A2	Single session	TUG the strongest single predictor of self-reported physical function (56% of variance; *p* < 0.05); knee-extension strength 0.60 Nm/kg and leg-extension power 0.91 W/kg, both far below healthy norms.
Rose MR (2001) [21]	Prospective cohort	11	A1	6 mo	Composite % predicted muscle strength declined ~4% over 6 mo (borderline, *p* = 0.05).
Cox (2011) [19]	Longitudinal case series	64	A1	Median 12 yr (IQR 11–13)	MMT −3.5 ± 1.6%/yr (28.8 ± 11.9% per 10 yr); QMT −5.4 ± 3.5%/yr (39.4 ± 21.8% per 10 yr); major end-stage disability but normal life expectancy.
Sangha (2021) [20]	Prospective cohort	181 (130 ≥1 follow-up)	A1	Median 1.3 yr (mean 2.0; range 0–7.3)	MMT −3.7%/yr (95% CI 3.1–4.3), MVICT −3.8%/yr (2.7–4.9), IBM-FRS −6.3%/yr (5.5–7.2); non-linear, steeper early.
Lindgren (2023) [26]	Population case series	6	A1	Median 11 yr (range 10–18)	Early-onset IBM severe; median survival 14 yr from diagnosis; strength decline 0.91 ± 0.2%/mo (correlated with time, *p* < 0.001).
Cortese (2013) [18]	Cross-sectional (1-yr)	51	A1, A2	12 mo (2 visits)	1-yr decline: MMT 5.2 ± 5.9%, IBM-FRS 13.8 ± 10.4%, quadriceps QMT 27.9 ± 15.9% (all *p* < 0.001).
Hogrel (2014) [17]	Prospective cohort	13	A1	48 mo	Composite QMT −32.3% over 4 yr (SRM 1.62, *p* = 0.002); knee extension ~50 ± 30% loss; wrist extension not significant (*p* = 0.064); MMT composite NS (*p* = 0.326).
Lindberg (2012) [22]	Retrospective cohort	66	A1	Mean 61.1 ± 37.7 mo	Overall strength decline −0.79 ± 0.49%/mo; untreated ‘natural course’ −1.03%/mo (~12%/yr); knee extension fastest −1.12 ± 0.62%/mo (*p* < 0.001 vs. other muscles).
Benveniste (2011) [25]	Retrospective cohort	140	A1	Followed 1990–2008	Slowly progressive but not lethal; wheelchair in 37.5% at median 14 yr from onset; male walking-aid transition HR 2.39 (95% CI 1.47–3.86).
Lowes L.P. (2013) [33]	Natural-history cohort (abstract)	100 (55 followed)	A1, A2	Mean 17.7 mo	Longitudinal (*n* = 55): knee extension −2%/mo, knee flexion −1.1%/mo; 2 min walk −1.2 m/mo; IBM-FRS −0.32 pts/mo; PROMIS −1.1 pts/mo; mean 5.4 ± 8.6 falls/yr (up to weekly); rate of decline independent of age at diagnosis.
Oh T.H. (2010) [32]	Cross-sectional (abstract)	9	A1, A2	Single session	MVIC ratios (95% CI): hip flexor:extensor 0.77 (0.52–1.02), knee extensor:flexor 0.80 (0.34–1.26), ankle dorsiflexor:plantarflexor 0.33 (0.15–0.52); predicted % of normal lowest for knee extensor 24%, ankle dorsiflexor 34%, hip flexor 39%; ankle-dorsiflexor asymmetry most marked (*p* = 0.025); anterior > posterior weakness.
Houston P. (2016) [75]	Natural-history cohort (abstract)	65 (of ~300 planned)	A1	Baseline (2-yr study)	Baseline (*n* = 65): age 70.2 ± 6.8 yr; 76.9% male; disease duration 11.5 ± 5.8 yr; dysphagia 65.6%; falls prior month 21.5%; nonambulatory 6.3%; sIFA 48.3 ± 20.6/100; quadriceps QMT 9.3 ± 10.4 (R)/9.7 ± 10.6 kg (L); SPPB 6.6 ± 2.7/12; 6MWD 302 ± 138 m.
**A2. Motor Performance (Gait & Transitional Tasks)**					
**Study**	**Design**	**IBM *n***	**Q(s)**	**Follow-Up**	**Principal Finding & Key Estimate (95% CI/SD/*p*)**
Bernhardt (2011) [28]	Cross-sectional	9	A2	Single session	Falling quadriceps strength → progressive knee hyperextension; knee position vs. hip-flexor strength R = 0.854 (*p* = 0.003), vs. maximum stance knee moment R = 0.902 (*p* = 0.001).
Davenport (2015) [29]	Cross-sectional	42	A2	2-day session	Lower-extremity MVIC ~27% of predicted (knee extensors 14.9%; SD reported); gait markedly slower than norms, worse in fast condition (all *p* ≤ 0.001).
Dunlap (2014) [27]	Retrospective cohort	53	A2, A3	Mean onset-to-visit 9.4 ± 0.8 yr	Knee-extension decline 21.8 ± 3.7%/yr (5-yr 68.6 ± 5.3%) vs. handgrip 3.5–7.2%/yr (*p* < 0.001); strength fell significantly as patients moved to more supportive gait devices.
**A3. Falls & Balance**					
**Study**	**Design**	**IBM *n***	**Q(s)**	**Follow-Up**	**Principal Finding & Key Estimate (95% CI/SD/*p*)**
Hiscock (2014) [34]	Cross-sectional survey	62 (86% response)	A3	Single survey	Falls near-universal: history 98% (61/62); ≥1 fall/3 mo 60% (37/62); fell in prior 3 mo 77% (48/62); major injury in 5% of 175 falls.
Nandakumar (2026) [36]	Cross-sectional survey	193 IBM (IIM cohort 470)	A3	Recall (since dx; past year)	High burden: falls since diagnosis 79.8%, past-year 57.0%; IBM vs. DM fall risk age-adjusted OR 2.04 (95% CI 1.53–2.57), mobility-aid use OR 3.1 (95% CI 1.98–4.94).
Saiki (2023) [38]	Case report	1	A3	1 yr (stable)	82-yr-old with ~10-yr progressive quadriceps weakness and recurrent falls initially attributed to aging; grip L 10.6/R 6.6 kg. Single case, no CI.
Danckworth (2014) [37]	Cross-sectional	8 sIBM (of 89)	A3	Prior 12 mo	sIBM falls/yr median 2.5 (IQR 0–6.5); fractures in 37.5% (3/8); myopathies with greater walking impairment had higher fall/fracture risk.
Hunn S. (2024) [35]	Cross-sectional (abstract)	62	A2, A3	Falls over prior 6 mo	*n* = 62 (62.9% male, age 69.9, disease duration 11.1 yr); 93.5% ambulatory; 40.3% ≥1 fall in prior 6 mo; falls most frequent at IBM-FRS 19–27; patients <19 could not perform TUG; FES-I correlated with TUG (r = 0.90, *p* < 0.001) and IBM-FRS (r = −0.82, *p* < 0.01).
**A4. Lower-Limb Sensory, Proprioceptive & Peripheral-Nerve Function**					
**Study**	**Design**	**IBM *n***	**Q(s)**	**Follow-Up**	**Principal Finding & Key Estimate (95% CI/SD/*p*)**
Arnardottir, Svanborg & Borg (2003) [39]	Analytical cross-sectional	9	A4	Single session	Abnormal somatosensory thresholds: vibratory abnormal in 5/9 and thermal in 4/9; mean vibratory thresholds significantly higher than controls (*p* < 0.05); raised heat-pain thresholds—interpreted as large-fiber peripheral-nerve affection.
Sturm et al. (2025) [40]	Cross-sectional (multimodal)	19	A4	Single session	Large-fiber neuropathy in 67% and small-fiber skin-biopsy pathology in 72% (corneal confocal microscopy 32%); quantitative sensory testing indicated predominantly large-fiber damage (61%).
**A5. Intervention Effects**					
**Study**	**Design**	**IBM *n***		**Q(s)**	**Principal In-Scope Finding**
Orsini (2009) [76]	Case report	1		A5	Muscle strength improved in several lower-limb groups after PNF program, while functional independence (FIM) remained unchanged/stable.
Cherin (2015) [47]	Case series	6		A5	All 6 cases showed improvement in muscle strength and resolution of dysphagia; for 2 patients, improvement persisted ~12 months before clinical exacerbation. SCIg deemed feasible and safe.
Johnson (2007) [62]	Non-randomized interventional	7		A5	Significant improvements in isometric strength in all muscle groups tested, maximal in hip flexors; walking and stair-climbing times improved in all patients; no significant change in serum CK.
Santos (2014) [67]	Non-randomized interventional	1		A5	After 12 weeks of BFRRT, MSTN mRNA decreased (down-regulated), while endogenous MSTN inhibitors follistatin, follistatin-like 3, and SMAD-7 increased (up-regulated).
Amato (2014) [51]	RCT	14		A5	Positive: single-dose bimagrumab significantly increased primary outcome right TMV vs. placebo at 8 weeks (+6.5%) and left TMV (+7.6%); LBM increased +5.7% vs. placebo at 8 weeks.
Jørgensen (2018) [65]	RCT	22		A5	No between-group effect on pf-SF-36 or objective physical function tests.
Nakajima (2021) [70]	Crossover RCT	24 (mixed cohort)		A5	HAL cybernic treatment significantly improved ambulatory function vs. hoist-only in the mixed cohort: 2MWT distance +10.066% (crossover treatment effect).
Suzuki (2020) [71]	Case series	3		A5	HAL therapy maintained/improved ambulation in sIBM. Case 1 showed marked improvement after first course (2MW 146%, 6 m speed 120%) sustained through 8 courses (133% and 130%).
Gualano (2010) [66]	Non-randomized interventional	1		A5	After 12 wk, improvements in 1RM leg press (+15.9%), timed up-and-go balance/mobility (+60%), and thigh cross-sectional area (+4.7%); all SF-36 subscales improved (18–7400%); HAQ unchanged.
Bernhardt (2011) [69]	Prospective SCO cohort (pre–post)	9 (6 completed)		A5	Stance-control knee orthosis (SensorWalk) worn ~6 months; braced vs. unbraced gait: with the SCO, walking velocity (*p* = 0.025) and cadence (*p* = 0.007) were lower and step width wider (*p* = 0.035), and peak swing-phase knee flexion decreased (*p* = 0.021). Objective gait effects were mixed; subjects with knee-extensor strength >30–40% of normal had more positive outcomes and reported greater stability and fewer falls.
Hanna (2019) [49]	RCT	251		A5	At week 52, 6MWD change from baseline did not differ between any bimagrumab dose and placebo; bimagrumab showed a good safety profile relative to placebo but did not improve 6MWD.
Benveniste (2021) [54]	RCT	44		A5	Primary endpoint not met: no between-group difference in maximal knee-extension strength (median difference 3.78%, 95% CI −10.61 to 17.31; *p* = 0.85); secondary outcomes favored sirolimus for 6 min walk distance (+11.37%, 95% CI 0.36–20.86; *p* = 0.009), forced vital capacity, HAQ-DI and thigh fat fraction; IBM-FRS unchanged.
Adler (2026) [57]	Open-label single-arm phase 1 trial	16		A5	Primary endpoint not met: PPARGC1A gene expression showed a non-significant trend toward increase after pioglitazone (*p* = 0.099).
Dalakas (2009) [53]	Open-label trial	13		A5	QMT total strength declined 14.9% over the 12-month natural-history period but only 1.9% at 6 months post-CAMPATH (a ~13% differential gain).
Goel (2022) [60]	First-in-human dose-escalation (abstract)	C1 + C2 (0.1, 0.5 mg/kg)		A5	Target-engagement/PD only (≤6 mo follow-up): CD8 + KLRG1+ depletion 46–96% (0.1 mg/kg) and 98–99% (0.5 mg/kg) within 24 h, recovery from Day 84; also depleted CD4 + KLRG1+ and CD8 + CD57 + (LGLs). No functional/mobility outcome reported.
Johannes S (1998) [77]	Case report	1		A5	During IVIg treatment, muscle strength continuously increased to normal and muscle atrophy improved.
Mendell (2016) [58]	RCT	6		A5	6MWT improved in treated group and declined in untreated group. Annualized (median 1-year) change: treated +56.0 m/year (+4.7 m/month) vs. untreated −25.8 m/year (−2.1 m/month), *p* = 0.01.
Tani (2023) [72]	Retrospective cohort (claims database)	424		A5	Longer daily rehabilitation (>1.0 h) was associated with significantly greater ADL (BI) improvement vs. shorter rehabilitation after IPTW adjustment (RR 1.37).
Soueidan SA (1993) [44]	Open-label trial	4		A5	In total, 3 of 4 patients showed significant functional improvement and objective strength increase after 2 monthly IVIg infusions; greatest improvement in proximal, less atrophic muscles.
Amato (1994) [45]	RCT	9		A5	No patient improved objectively on AMS; in 6 of 9, the AMS declined despite IVIG. Disability score worsened in one patient (no. 8) while AMS was unchanged; unchanged in the other 8.
Dalakas MC (1997) [41]	Crossover RCT	22		A5	Primary outcome negative: IVIg did not differ significantly from placebo in overall muscle strength. During IVIg, patients gained a mean of 4.2 MRC points.
Walter (2000) [42]	Crossover RCT	22		A5	Overall improvement or stabilization in 90% of patients over the study (no disease progression), contrasting expected decline in untreated s-IBM.
Dalakas (2001) [43]	RCT	37		A5	No significant change in muscle strength (QMT or MRC) between groups at months 2, 3, or 4; only small, insignificant losses (minor non-significant gains in first month in IVIg group, not sustained).
Arnardottir S (2003) [63]	Uncontrolled pre-post pilot	7		A5	Muscle strength (MRC) and function (FI) not significantly improved after exercise, but none deteriorated (interpreted as possible prevention of expected disease/inactivity-related strength loss).
Dobloug (2012) [46]	RCT	16		A5	IVIG had short-term but non-sustained benefits. 2/14 IVIG patients (P13, P16) improved >20% in MMT total and ≥2 gradients in MMT quadriceps early in treatment, but not sustained.
Recher (2012) [78]	Case report	1		A5	Rapid subjective improvement in muscle strength within days of the first low-dose IVIG infusion, with a trend to objective improvement over subsequent months.
Cherin (2016) [48]	Case series	7		A5	Whole cohort: Slight non-significant improvement in muscle strength (10/14, 71.1% improved) and statistically significant improvement in muscle disability (7/11, 63.6% improved).
Wallace (2019) [68]	Crossover RCT	17		A5	IBM group improved aerobic capacity with training, with a strong effect size.
Pawlitzki (2022) [55]	Case report	1		A5	Rapid and sustained clinical improvement in motor deficits after starting sirolimus: MMT8 rose from 99/150 to 123/150 at 9 months and 136/150 at ~12 months; IBM-FRS improved from 19/40 to 29/40.
Machado (2023) [52]	RCT	152		A5	Arimoclomol showed no benefit: at month 20, IBM-FRS declined in both groups with no significant treatment difference (mean difference −0.99, favoring placebo, *p* = 0.12).
Badrising (2025) [56]	RCT—protocol (no results)	140 (planned)		A5	No trial results reported—this is a protocol/design paper.
Spector (1997) [61]	Non-randomized interventional	5		A5	Dynamic 3RM strength improved in the least-weakened muscles with group mean increases of 25–120% after 12 weeks; significant increases in 5 of 8 exercises.
D’Alton (2022) [64]	Case report	1		A5	Training was safe and appeared to maintain (not improve) muscle strength; no significant change (improvement or deterioration) in isokinetic strength, with variable responses between muscle actions.
Connor (2023) [59]	Crossover RCT	14		A5	No significant testosterone-associated improvement in quadriceps extension strength, lean body mass, or any secondary physical/functional outcome vs. placebo over 12 weeks.
Sivakumar (2020) [50]	Open-label extension trial	10		A5	Good safety profile and well tolerated over 2 years; no new safety signals or deaths.
Machado (2013) [73]	RCT (proof-of-concept)	24 (16 vs. 8)		A5	Arimoclomol vs. placebo, 4 mo treatment (f/u 12 mo): safe and well tolerated (AEs 6.8 vs. 6.5/pt; 1 SAE, post-biopsy hypertension); exploratory efficacy showed numerically slower decline, near-significant at 8 mo (IBM-FRS *p* = 0.055; right-grip MVICT *p* = 0.064); no significant difference at 12 mo (IBM-FRS change −2.03 ± 2.68 vs. −3.50 ± 3.35, *p* = 0.538) or in MVICT sum, DEXA fat-free mass, or muscle HSP70.
Kenya M. (2015) [79]	Retrospective cohort (abstract)	6 IVIg vs. 10 untreated		A5	Long-term IVIg (400 mg/kg/d ×5 d/course, q2–6 mo; mean 6.9 yr, ~25 courses): median onset-to-assistive-device 10 vs. 7 yr and onset-to-wheelchair 15 vs. 9 yr (IVIg vs. untreated); significantly slower progression of walking disability with IVIg (Kaplan–Meier, log-rank).
Liang C. (2019) [74]	Matched-control pilot (abstract)	7 (4 exercise, 3 control)		A5	Lower-limb BFR (50%, 20–30% RM, 3×/wk; 12 wk + 4-wk f/u): muscles with MRC ≥2 exercisable; reps/weights increased by wk 4 with a trend to continued gain over 4 mo; no concerning adverse events. Safe, possibly helpful.

Number of reports versus studies: The 64 studies include 56 full peer-reviewed reports and 8 conference abstracts. When a trial or cohort generated multiple publications, such as pharmacological programs or the blood-flow-restriction trial with its substudy and case reports, these were consolidated into a single study. As a result, the study count is lower than the total number of source publications. Sample size versus unique participants: Summing the enrolled IBM participants across all full reports yields an aggregate of approximately 2457. However, this figure does not represent unique individuals. Several natural-history cohorts originate from the same specialist centers, and some patients contribute data to more than one study. Therefore, the actual number of unique IBM participants is substantially lower and cannot be determined precisely from the published reports. Protocol-only (planned-sample) and mixed-cohort abstract records are excluded from this aggregate. Additionally, abstracts often did not report an analyzable IBM sample size (*n* = NR).

**Table 2:** (**a**) Pharmacological intervention studies in IBM. Comparator is placebo unless stated. *n* = IBM participants analyzed. Effect estimates are between-group unless the design is uncontrolled (within-group change reported). (**b**) Dedicated rehabilitation intervention table—exercise and training studies in IBM. Effect estimates are between-group where a comparator existed; single-arm and *n* = 1 studies report within-subject change only and carry no confidence interval. (**c**) Assistive-device and robotic studies in IBM. *n* = IBM participants; several device trials enrolled mixed neuromuscular cohorts in which IBM was a minority, so no IBM-specific effect estimate is available.

**(a)**									
**Study**	**Agent**	**Design**	***n***	**Comparator**	**Follow-Up**		**Outcome & Effect Estimate (95% CI)**		**Safety**
Amato (2014) [51]	Bimagrumab	RCT	14 (10 to extension)	Placebo	24 wk (primary wk 8)		Positive at wk 8: right thigh-muscle volume (TMV) +6.5% and left +7.6% vs. placebo (LS-mean treatment effect); 6MWD +14.6% at wk 16, +5.7% at wk 24. CIs not reported for the primary contrast.		No drug-related serious AEs.
Hanna (2019 [49], RESILIENT)	Bimagrumab	RCT	251	Placebo	52 wk		Primary not met: 6MWD LS-mean difference vs. placebo +17.6 m (SE 14.3, 10 mg/kg), +18.6 m (SE 14.2, 3 mg/kg), −1.3 m (SE 14.1, 1 mg/kg); no dose differed significantly.		≥1 AE ~100% all arms; two deaths; discontinuation for AEs 6% vs. 2%.
Benveniste (2021) [54]	Sirolimus	RCT	44 (ITT)	Placebo	12 mo		Primary (max knee-extension strength) not met: median difference 3.78% (95% CI −10.61 to 17.31), *p* = 0.85. Secondary 6MWD favored sirolimus: +11.37% (95% CI 0.36 to 20.86), *p* = 0.009.		Serious AEs: 10/22 (45%) sirolimus vs. 6/22 (27%) placebo; 4/22 stopped drug.
Machado (2023) [52]	Arimoclomol	RCT	152 (134 completed)	Placebo	20 mo		Primary not met: IBM-FRS change difference −0.99 favoring placebo, *p* = 0.12; 6MWT difference −1.79 m; hand-grip −1.11 kg.		≥1 AE 94%; more events on arimoclomol (593 vs. 450).
Connor (2023) [59]	Testosterone	Crossover RCT	14	Placebo	26 wk (2 × 12-wk arms)		No benefit: quadriceps extension mean difference 4.69 Nm at 30° (95% CI −5.68 to 11.46), *p* = 0.67; total lean body mass +0.30 kg (95% CI −0.77 to 1.38), *p* = 0.61.		22 falls across crossover; no drug-attributable serious AEs.
Dalakas (1997) [41]	IVIg	Crossover RCT	22 (19 analyzed)	Placebo	3 mo/arm (+3 mo follow-up)		Not significant: IVIg +4.2 MRC points (range −16 to +39.8) vs. placebo −2.7; crossover placebo → IVIg +5.7 vs. IVIg → placebo −4.5. No CI reported.		Mild headache; one with more severe headache.
Dalakas (2001) [43]	IVIg + prednisone	RCT	37 (36 completed)	Placebo	3 mo (+1 mo follow-up)		No between-group difference in QMT/MRC; lower-extremity MRC change +11.5 ± 26.8% (IVIg) vs. +6.8 ± 15.3% (placebo). No CI reported.		No serious AEs in IVIg arm; one death (placebo, post-infusion).
Amato (1994) [45]	IVIg	RCT	9	Placebo	~3–4 mo		No objective improvement on average manual score (AMS); AMS declined in 6/9; disability worsened in one (no. 8). No CI (individual data only).		Neutropenia curtailed one; headache limited one.
Dobloug (2012) [46]	IVIg	RCT	16 (serial MMT 14/16)	Placebo	Mean 23 mo (range 11–48)		Short-term, non-sustained benefit; CK fell >20% in 7/16. No between-group effect estimate/CI reported.		Mild–moderate infusion headaches (led to dose reduction).
Walter (2000) [42]	IVIg	Crossover RCT	22 (20 completed)	Placebo	12 mo (+ext. mean 15.7 mo)		Within-group MRC +4.9 ± 6.4% (median 2.8%), *p* = 0.003; improvement/stabilization in 90%, *p* = 0.001. Between-group estimate not reported (uncontrolled improvement claim).		No serious side effects.
Mendell (2016) [58]	Follistatin gene therapy	RCT	6	No treatment (untreated comparator)	Median 14 mo (range 2–24)		6MWT treated median +62.5 m (annualized +56 m/yr) vs. decline in untreated (*n* = 8). No CI reported.		No treatment-related AEs/SAEs.
Adler (2026) [57]	Pioglitazone	Open-label	16 (13 initiated)	None (uncontrolled)	48 wk		Primary not met: PPARGC1A expression mean difference 1.19 (95% CI −0.26 to 2.64), *p* = 0.099 (non-significant trend).		AEs attributed to therapy in 2/13 (15%): one myalgia, one headache.
Dalakas (2009) [53]	Alemtuzumab	Open-label	13	None (self as natural-history control)	12 mo (safety to 2 yr)		QMT −1.9% at 6 mo vs. −14.9% pre-treatment natural-history decline (~13% differential); 4/13 gained mean ~10%. No CI (single-arm).		Well tolerated; transient infusion hypotension ×3.
Sivakumar (2020) [50]	Bimagrumab (extension)	Open-label	10	None (uncontrolled)	Up to 104 wk		TMV +4.5% (wk 16); lean body mass peak +7.5% (wk 24); no controlled functional benefit sustained. No CI reported.		100% ≥1 AE; 3 discontinued for serious AEs (MI, carcinoma, dementia).
Soueidan (1993) [44]	IVIg	Open-label	4	None (uncontrolled)	6–9 mo		Cumulative MRC rose in 3/4 (e.g., Pt1 97 → 112). No CI (single-arm, *n* = 4).		None reported.
Cherin (2015) [47]	SCIg	Case series	6	None (uncontrolled)	4.5–27 mo		Muscle-power score (/88) improved in 4/5 followed (e.g., case 4 66 → 81; case 6 61 → 71); case 2 declined 64 → 56 at 18 mo. No CI.		No serious AEs; injection-site swelling/pain (case 2).
Cherin (2016) [48]	SCIg	Case series	7 (cohort 14)	None (uncontrolled)	Mean 18.8 mo (range 4.5–42)		IBM subgroup muscle strength +3.85 points; muscle disability score −5 points (uncontrolled within-group). No CI.		No serious AEs over 1215 injections.
Pawlitzki (2022) [55]	Sirolimus	Case report	1	None (uncontrolled)	24 mo		MMT8 99 → 136/150 at 12 mo; IBM-FRS 19 → 29/40. Single case, no CI.		Well tolerated; no AEs.
Johannes (1998) [77]	IVIg	Case report	1	None (uncontrolled)	28 mo		MRC grade 4 → 5 (normal) over 28 mo. Single case, no CI.		None reported.
Recher (2012) [78]	Low-dose IVIg	Case report	1	None (uncontrolled)	12 mo		CK normalized within 1 mo; clinical remission sustained to 12 mo. Single case, no CI.		No IVIg-related AEs.
Goel (2022) [60]	ABC008 (anti-KLRG1)	First-in-human dose-escalation (abstract)	C1 + C2 (0.1, 0.5 mg/kg)	None (uncontrolled)	≤6 mo		Immunological/PD only: max CD8 + KLRG1+ depletion 46–96% (0.1 mg/kg) and 98–99% (0.5 mg/kg) within 24 h; recovery from Day 84. No functional/mobility outcome reported.		NR (early safety/PD study).
Badrising (2025) [56]	Sirolimus (OPTIMISM)	RCT (protocol only)	140 (planned)	Placebo	84 wk (planned)		No results—protocol/design paper (assumed placebo IBM-FRS decline ~2.8/yr; target detectable difference 3.0 points).		To be assessed as a secondary safety objective.
Machado (2013) [73]	Arimoclomol	RCT (proof-of-concept, full report)	24 (16 arimoclomol, 8 placebo)	Placebo	4-mo treatment; f/u to 12 mo		Exploratory efficacy: decline numerically slower with arimoclomol; near-significant at 8 mo for IBM-FRS (*p* = 0.055) and right-grip MVICT (*p* = 0.064); IBM-FRS change at 12 mo −2.03 ± 2.68 vs. −3.50 ± 3.35 (placebo), *p* = 0.538; no significant difference in MVICT sum, DEXA fat-free mass, or muscle HSP70.		Well tolerated; AEs 6.8 vs. 6.5/patient; 1 SAE (post-biopsy hypertension); no ocular toxicity or arrhythmia.
Kenya (2015) [79]	IVIg	Case series (abstract)	6 IVIg vs. 10 untreated	Untreated observation	Mean 6.9 ± 3.3 yr		Median onset-to-assistive-device 10 vs. 7 yr and onset-to-wheelchair 15 vs. 9 yr (IVIg vs. untreated); significantly slower progression of walking disability with IVIg (log-rank).		NR.
**(b)**									
**Study (Design; *n*)**	**Type**	**Frequency & Session Time**	**Intensity**	**Progression**	**Supervision**	**Comparator**	**Adherence**	**Outcome & Effect Estimate (95% CI)**	**Safety**
Wallace (2019) [68]; randomized crossover, assessor-blind; *n* = 17 (16 completed)	Aerobic cycle-ergometer training	3×/week; up to 30 min/session; 12 weeks (36 sessions)	60 → 70 → 80% VO_2_peak (heart-rate targeted); Borg scale for weaker participants	Intensity stepped up at weeks 4 and 8	Community-gym fitness instructors plus research physiotherapist (visits + telephone every 2 weeks)	Within-subject 12-week control period (8-week washout)	91% of sessions completed	VO_2_peak +17.4% with training vs. −1.3% with control; effect size Cohen d = 1.72 (SD 0.43); work rate +17.3%. Other secondary measures (strength, walking, QoL) unchanged.	No CK rise; feasible and safe; no serious AEs.
Jørgensen (2018) [65]; exploratory RCT; ITT *n* = 22 (per-protocol *n* = 19)	Low-load blood-flow-restricted (BFR) resistance (leg press, knee ext/flex, calf)	2×/week; 12 weeks; 3 sets × 25RM (4 sets from week 9)	~20% 1RM; pneumatic cuff 110 mmHg	Sets increased at week 9; knee flexion added week 4	One exercise physiologist supervised all sessions	Non-exercising control group (*n* = 11)	Per-protocol ≥66% compliance; one excluded (37%)	Knee-extensor strength preserved vs. control (control −9.2%, *p* = 0.023; BFR +0.9%; per-protocol between-group +15.0%, *p* = 0.026); IBM-FRS favored BFR (ITT between-group difference 5.8%, *p* = 0.018); pf-SF-36 and objective function not significant.	Two BFR dropouts attributed to intervention (severe fatigue; fall from fatigue, no injury); no accelerated disease activity.
Arnardottir (2003) [63]; uncontrolled pre-post pilot; *n* = 7 (dynamometry 4/7)	Home resistive exercise + self-paced walk	5 days/week; 15 min exercise + 15 min walk; 12 weeks	Moderate or easy version; weight cuffs 0.25–2 kg; 10 repetitions	None able to increase load after 6 weeks	Tape-recorded instructions, weekly telephone; one assisted by physiotherapist	None (single-arm; 2 non-diseased biopsies as histological controls only)	Well tolerated; all completed (one assisted); exercise diary	MRC and functional index not significantly changed but none deteriorated (interpreted as prevention of expected decline); isokinetic torque and CK unchanged; endothelial EN-4^+^ area and IL-1Ra^+^ cells fell (*p* < 0.05). No CI.	No harm; no clinical deterioration or increased inflammation.
Spector (1997) [61]; uncontrolled pre-post pilot; *n* = 5	Progressive resistance (knee ext/flex; elbow & wrist flexion)	3×/week (non-consecutive days); 12 weeks (36 sessions); 3 sets (10/15/20 reps)	Initial concentric ~5RM (pneumatic load)	Load readjusted weekly	Supervised	None (single-arm, within-subject before/after)	36 sessions implied; not quantified	Dynamic 3RM strength +25–120% (significant in 5/8 exercises; largest in least-affected muscles; means ± SE, e.g., left knee curl +106 ± 19%); isometric torque and cross-sectional area not significant; MRC changed ≤½ grade. No CI.	No AEs; CK, immune and histology markers unchanged.
Johnson (2007) [62]; uncontrolled pre-post; *n* = 7	Individualized home functional exercise	Daily; 16 weeks; session time NR	Mild	NR	Home program (self-directed)	None (single-group)	“Well tolerated”; not quantified	Isometric strength improved in all groups tested (maximal in hip flexors); walking and stair-climbing times improved in all patients. No effect size/CI reported.	CK unchanged; well tolerated.
Tani (2023) [72]; observational cohort (IPTW-adjusted); *n* = 424	Inpatient multidisciplinary rehabilitation (PT/OT/ST); exposure = daily rehab dose	Longer (>1.0 h/day) vs. ≤1.0 h/day; overall mean 52.8 min/day (SD 22.8); in-hospital ≤60 days	Not graded (dose defined by duration)	Threshold dose-response tested at 1.5 h and 2.0 h	Inpatient supervised rehabilitation	Internal comparator: ≤1.0 h/day group (*n* = 265)	NR	ADL (Barthel) improvement RR 1.37 (95% CI 1.06–1.78); toileting RR 2.57 (1.66–4.12); mobility RR 2.01 (1.38–3.00); dose-response to RR 3.98 (3.06–5.02) at 2.0 h.	Not reported.
D’Alton (2022) [64]; case report; *n* = 1	Supervised resistance (isometric + isotonic; resistance bands)	3×/week; 60 min/session; 16 weeks (two 8-week blocks)	Moderate; RPE maintained 7–8/10	Adjusted when too easy/difficult	Two biokineticists	None (single-subject)	Tolerated and complied well	Isokinetic strength maintained (no significant change, variable by action); self-reported fatigue improved; Barthel unchanged. Single case, no CI.	No AEs beyond limb stiffness; CK minimal (188/181 IU/L).
Gualano (2010) [66]; case report; *n* = 1	Resistance + vascular occlusion/BFR (leg ext, leg press, half-squat)	2×/week; 12 weeks; 3 sets × 15RM	50% occlusion pressure (~65 mmHg); moderate load	Progressed to keep at ≤15RM; 10-week detraining phase added	≥2 investigators supervised	None (single-subject; historical self-comparison)	Completed 12 weeks; rate NR	1RM leg press +15.9%; timed-up-and-go +60%; thigh cross-sectional area +4.7%; strength retained after 10-week detraining (−0.5%). Single case, no CI.	No disease flare; no pain, soreness, or vascular complication.
Santos (2014) [67]; case report (molecular substudy); *n* = 1	BFR resistance (leg press, knee ext, half-squat)	2×/week; 12 weeks; 3 sets × 15RM	50% total occlusion pressure; light load	Progressed to ≤15RM	Two physical trainers	None (single-subject)	Monitored; rate NR	Myostatin mRNA −25%, follistatin +40%, follistatin-like-3 +70% (attenuated myostatin signalling); prior report of same case: leg-press 1RM +11.6%, function +60%, thigh CSA +4.7%. Single case, no CI.	Only mild acute pain; no other AEs.
Orsini (2009) [76]; case report; *n* = 1	Home proprioceptive-neuromuscular-facilitation (PNF) functional exercise	Daily; 40–50 min/session; 16 weeks (100 sessions)	Mild; 3 sets × 10 repetitions per diagonal	Not described	Delivered by one physiotherapist	None (single-subject pre/post)	Completed 100 sessions; no formal metric	Lower-limb MRC improved (iliopsoas, quadriceps, glutei, adductors, plantarflexors each 3 → 4; tibialis anterior 0 unchanged); functional independence (FIM) stable. Single case, no CI.	No pain or discomfort; no AEs.
Liang (2019) [74]; conference abstract (matched-control pilot); *n* = NR	Light-load BFR resistance	3×/week; 12 wk + 4-wk f/u	20–30% RM; 50% BFR	NR	NR	Matched controls (*n* = 3)	NR	Muscles with MRC ≥2 exercisable; all increased reps/weights by wk 4 with a trend to continued improvement over 4 mo; hand-held dynamometry unreliable while 2MWT/TUG/MCHSAT more stable.	No concerning adverse events.
**(c)**									
**Study**	**Device**	**Design (IBM *n*)**		**Protocol & Comparator**	**Follow-Up**		**Outcome & Effect Estimate (95% CI)**		**Safety**
Nakajima (2021) [70]	Robotic Hybrid Assistive Limb (HAL), lower-limb	Open-label crossover RCT; mixed 8-disease cohort (1 IBM)		40 min sessions (20–30 min walking), 9 sessions/block, ≤4×/week over 13 weeks × 2 blocks; comparator hoist-only walking	13 weeks/block + 4-week post-observation		Whole-cohort 2MWT +10.066% (95% CI 0.67 to 19.46), *p* = 0.037; 10MWT cadence +7.1% (95% CI 2.61 to 11.59); total MMT +3.04, *p* = 0.039. No separate IBM (*n* = 1) efficacy estimate.		All AEs mild and remediable; no serious or device-failure AEs.
Suzuki (2020) [71]	Robotic HAL, lower-limb	Case series; *n* = 3 IBM (inpatient)		3×/week × 3 weeks per course, 20 min/session; 2–8 courses; no comparator	Case 1 30 mo; cases 2–3 ~1 yr		Ambulation maintained/improved: Case 1 2 min walk 56.7 → 75.8 m (146% after first course, 133% after eighth); cases 2–3 ~90–107%. No CI (*n* = 3).		Case 1 fell and fractured a rib; authors flag increased fall risk.
Bernhardt (2011) [69]	Stance-control knee orthosis (SensorWalk)	Prospective pre–post cohort; *n* = 9 (6 completed)		~6 months of home SCO use; within-subject braced vs. unbraced gait analysis (no separate control group)	6 months		With the SCO: velocity lower (*p* = 0.025), cadence lower (*p* = 0.007), step width wider (*p* = 0.035), peak swing-phase knee flexion reduced (*p* = 0.021); objective gait effects mixed, benefit greatest in subjects with knee-extensor strength >30–40% of normal, who reported improved stability and fewer falls.		No adverse events reported; participants complained of device size, weight, and donning difficulty.

**Table 3:** (**a**) Risk of bias in randomized trials (RoB 2, outcome level). (**b**) Risk of bias in non-randomized intervention studies (ROBINS-I). (**c**) Risk of bias in prognostic/natural-history cohorts (QUIPS). (**d**) Methodological quality of analytical cross-sectional studies (JBI). (**e**) Methodological quality of case series/case reports (JBI). (**f**) Conference abstracts (gray literature—not formally appraisable).

**(a)**								
**Study**	**Randomization**	**Deviations**		**Missing Data**	**Outcome Meas.**	**Reported Result**		**Overall**
Amato (2014) [51]	Low	Low		Low	Low	Low		Low
Jørgensen (2018) [65]	Some concerns	Some concerns		Low	High	Low		High
Nakajima (2021) [70]	Low	Some concerns		High	Some concerns	Low		High
Hanna (2019) [49]	Low	Low		Low	Low	Low		Low
Dalakas MC (1997) [41]	Low	Some concerns		Some concerns	Low	Some concerns		Some concerns
Walter (2000) [42]	Some concerns	Low		Low	Low	Some concerns		Some concerns
Dalakas (2001) [43]	Low	Low		Some concerns	Low	Some concerns		Some concerns
Wallace (2019) [68]	Low	Some concerns		Some concerns	Some concerns	Low		Some concerns
Machado (2023) [52]	Low	Low		Low	Low	Low		Low
Badrising (2025) [56]	Low	Some concerns		Some concerns	Some concerns	Low		Some concerns
Connor (2023) [59]	Low	Some concerns		Low	Low	Low		Some concerns
Benveniste (2021) [54]—sirolimus	Low	Some concerns		Some concerns	Low	Some concerns		Some concerns
Machado (2013) [73]	Some concerns	Low		Low	Low	Some concerns		Some concerns
**(b)**								
**Study**	**Confounding**	**Selection**	**Classification**	**Deviations**	**Missing Data**	**Outcome Meas.**	**Reported Result**	**Overall**
Johnson (2007) [62]	Serious	Moderate	Low	Moderate	Moderate	Serious	Moderate	Serious
Adler (2026) [57]	Serious	Moderate	Low	Moderate	Moderate	Low	Moderate	Serious
Dalakas (2009) [53]	Serious	Moderate	Low	Serious	Low	Serious	Moderate	Serious
Mendell (2016) [58]	Critical	Serious	Low	Serious	Moderate	Serious	Moderate	Critical
Tani (2023) [72]	Serious	Moderate	Moderate	Moderate	Moderate	Moderate	Moderate	Serious
Soueidan SA (1993) [44]	Serious	Moderate	Low	Serious	Low	Serious	Moderate	Serious
Amato (1994) [45]	Serious	Moderate	Low	Moderate	Moderate	Serious	Low	Serious
Arnardottir S (2003) [63]	Serious	Moderate	Low	Moderate	Serious	Serious	Moderate	Serious
Dobloug (2012) [46]	Serious	Serious	Moderate	Serious	Serious	Moderate	Moderate	Serious
Cherin (2016) [48]	Serious	Moderate	Low	Moderate	Serious	Serious	Moderate	Serious
Spector (1997) [61]	Serious	Moderate	Low	Low	Low	Moderate	Moderate	Serious
Sivakumar (2020) [50]	Serious	Moderate	Low	Moderate	Serious	Moderate	Moderate	Serious
**(c)**								
**Study**	**Participation**	**Attrition**	**Factor Meas.**	**Outcome Meas.**	**Confounding**	**Analysis**		**Overall**
Allenbach (2012) [16]	Moderate	High	Low	Low	Moderate	Moderate		Moderate
Oldroyd (2020) [24]	Moderate	High	Moderate	Moderate	Moderate	Moderate		High
Rose MR (2001) [21]	Moderate	High	Moderate	Low	Moderate	Moderate		Moderate
Cox (2011) [19]	Low	High	Low	Moderate	Moderate	Moderate		High
Sangha (2021) [20]	Moderate	High	Moderate	High	Moderate	Low		Moderate
Lindgren (2023) [26]	Low	Moderate	Low	Low	High	Moderate		High
Cortese (2013) [18]	Moderate	High	Moderate	Low	Moderate	Moderate		High
Hogrel (2014) [17]	Moderate	High	Low	Low	Moderate	Moderate		High
Lindberg (2012) [22]	Moderate	Moderate	Low	Low	High	Moderate		Moderate
Benveniste (2011) [25]	Moderate	Low	Moderate	Moderate	High	Moderate		Moderate
**(d)**								
**Study**	**Selection**	**Exposure Meas.**	**Outcome Meas.**		**Confounding**	**Analysis**		**Overall**
Bernhardt (2011)—gait [28]	Moderate	Low	Low		Moderate	Moderate		Moderate
Lowes (2012) [30]	Moderate	Low	Moderate		Moderate	Moderate		Moderate
Davenport (2015) [29]	Moderate	Low	Moderate		Moderate	Moderate		Moderate
Jørgensen (2017) [31]	Moderate	Low	Moderate		Moderate	—		Moderate
Hiscock (2014) [34]	Moderate	Moderate	High		Moderate	—		Moderate
Bernhardt (2011)—orthosis [69]	High	Low	High		Moderate	—		High
Dunlap (2014) [27]	Moderate	Moderate	High		Moderate	—		High
Nandakumar (2026) [36]	High	High	High		Moderate	—		High
Danckworth (2014) [37]	High	High	High		Moderate	—		High
Arnardottir, Svanborg & Borg (2003) [39]—A4	Moderate	Low	Low		High	Moderate		Moderate
Sturm (2025) [40]—A4	Moderate	Low	Moderate		High	Moderate		Moderate
**(e)**								
**Study**	**Selection**	**Ascertainment**	**Outcome Meas.**	**Attribution**	**Follow-Up**	**Analysis**		**Overall**
Orsini (2009) [76]	High	Low	Moderate	High	Low	High		High
Cherin (2015) [47]	High	Low	High	High	High	—		High
Santos (2014) [67]	High	High	Low	Moderate	Low	Moderate		High
Suzuki (2020) [71]	High	Low	High	High	High	—		High
Gualano (2010) [66]	High	Low	Moderate	High	High	—		High
Johannes S (1998) [77]	High	Low	Moderate	High	—	—		High
Saiki (2023) [38]	Low	Low	Moderate	High	—	—		High
Recher (2012) [78]	Low	Low	High	Low	High	—		High
Pawlitzki (2022) [55]	High	Low	Moderate	High	Moderate	—		High
D’Alton (2022) [64]	Moderate	Low	Low	High	Moderate	—		High
**(** **f** **)**								
**Study**		**Review Question**			**Status**			
Lilleker (2019) [23]		A1			Not formally appraisable (abstract)			
Goel (2022) [60]		A5			Not formally appraisable (abstract)			
Lowes L.P. (2013) [33]		A1/A2			Not formally appraisable (abstract)			
Hunn S. (2024) [35]		A3			Not formally appraisable (abstract)			
Oh T.H. (2010) [32]		A1/A2			Not formally appraisable (abstract)			
Houston P. (2016) [75]		A1			Not formally appraisable (abstract)			
Kenya M. (2015) [79]		A5			Not formally appraisable (abstract)			
Liang C. (2019) [74]		A5			Not formally appraisable (abstract)			

Ratings: RoB 2—low/some concerns/High. ROBINS-I—low/moderate/serious/critical. QUIPS and JBI—low/moderate/high risk. NA = domain not applicable. Full written justifications for every domain are in the accompanying risk-of-bias workbook.

**Table 4:** (**a**) Evidence profile—pharmacological interventions in IBM (patient-important benefits and harms). Population: adults with IBM. Comparator: placebo unless stated. (**b**) Evidence profile—non-pharmacological interventions in IBM. (**c**) Evidence profile—natural history/progression (A1), motor performance (A2), falls burden (A3), and lower-limb sensory/peripheral-nerve function (A4). Prognostic/association framework; ratings describe certainty in the estimated rate or association. (**d**) Evidence gaps (concepts searched but returning no eligible IBM study—not rated). Listed concepts were part of the direct-IBM search (arms A3, A5a, and the proprioceptive/neurological arm A4; Appendix A). Sensory/peripheral-nerve function is not a gap—it is measured directly and synthesized under A4 (Section 3.4)—but proprioceptive acuity and posturography remain unmeasured.

**(a)**						
**Intervention · Outcome**	**Studies (Participants)**	**Design**	**Effect Estimate**	**Follow-Up**	**Certainty**	**Reasons**
IVIg · muscle strength (MRC/QMT)	3 RCTs (*n* ≈ 77)	Randomized crossover/parallel	No significant difference vs. placebo (e.g., +4.2 vs. −2.7 MRC points, ns)	3–12 mo	Low	↓ Risk of bias (small crossover trials, incomplete blinding detail); ↓ imprecision (few participants, wide CIs)
IVIg · adverse events (harm)	3 RCTs + series	RCT/observational	Mild headache; no excess serious events	3–12 mo	Low	↓ imprecision
Bimagrumab · 6 min walk distance	1 phase 2b RCT (*n* = 251) + extension (*n* = 10)	RCT	LS mean difference 17.6 m (99% CI −19.6 to 54.8), *p* = 0.22—no benefit	52 wk	Moderate	↓ imprecision (single trial; CI spans no effect)
Bimagrumab · adverse events (harm)	1 RCT (*n* = 251)	RCT	Any AE ~100%; falls, muscle spasms, diarrhoea more frequent	52 wk	Moderate	↓ imprecision
Arimoclomol · IBM-FRS progression	1 phase 2/3 RCT (*n* = 152)	RCT	Mean difference −0.99 (favoring placebo), *p* = 0.12—no benefit	20 mo	Moderate	↓ imprecision
Arimoclomol · adverse events (harm)	1 RCT (*n* = 152)	RCT	More AEs on arimoclomol (99% vs. 90%)	20 mo	Moderate	↓ imprecision
Sirolimus · maximal knee-extension strength (primary)	1 phase 2b RCT (*n* = 44); phase III OPTIMISM ongoing	RCT	No difference: median difference 3.78% (95% CI −10.61 to 17.31), *p* = 0.85	12 mo	Low	↓ imprecision (single small trial, *n* = 44); ↓ inconsistency (negative primary vs. positive secondary/post hoc endpoints)
Sirolimus · 6 min walk distance (secondary)	1 phase 2b RCT (*n* = 44)	RCT	Favored sirolimus: between-group difference +11.37% (95% CI 0.36–20.86), *p* = 0.009; FVC, HAQ-DI and thigh fat fraction also favored sirolimus	12 mo	Very low	Secondary/post hoc outcome; ↓ imprecision; ↓ indirectness (surrogate for mobility)
Sirolimus · adverse events (harm)	1 phase 2b RCT (*n* = 44)	RCT	Serious AEs 10/22 (45%) vs. 6/22 (27%) placebo; 4/22 (18%) discontinued	12 mo	Low	↓ imprecision
Alemtuzumab · strength	1 uncontrolled trial (*n* = 13)	Non-randomized	Transient slowing of decline, not sustained	12 mo	Very low	Non-randomized start; ↓ risk of bias (no control); ↓ imprecision
Follistatin gene therapy · 6 min walk distance	1 open-label trial (*n* = 6)	Non-randomized	+56 m/yr treated vs. −25.8 m/yr untreated, *p* = 0.01	1 yr	Very low	Non-randomized, uncontrolled, very small
Testosterone · quadriceps strength	1 crossover RCT (*n* = 14)	RCT	Mean difference 4.69 N·m (95% CI −5.68 to 11.46), *p* = 0.67—no benefit	12 wk	Low	↓ imprecision (very small); ↓ risk of bias
Pioglitazone/ABC008 · function	single early-phase (*n* ≈ 13/N NR)	Non-randomized phase 1	No patient-important functional outcome demonstrated	8 mo/SAD-MAD	Very low	Early-phase, mechanistic/safety endpoints only
**(b)**						
**Intervention · Outcome**	**Studies (Participants)**	**Design**	**Effect Estimate**	**Follow-Up**	**Certainty**	**Reasons**
Conventional resistance exercise · muscle strength/function	3 uncontrolled pre-post (*n* = 5, 7, 7) + 1 case	Non-randomized	Strength gains in least-affected muscles; no deterioration; no controlled effect	12 wk–16 wk	Very low	Non-randomized start; ↓ risk of bias (no control); ↓ imprecision
Conventional resistance exercise · safety (harm)	4 studies	Non-randomized	No adverse muscle effects; CK unchanged	12–16 wk	Low	Consistent safety signal
Aerobic training · aerobic capacity (VO_2_ peak)	1 crossover RCT (*n* = 17 IBM)	RCT	+17.4% training vs. −1.3% control; within-group Cohen d = 1.72	12 wk	Low	↓ imprecision (single small trial); ↓ indirectness (aerobic capacity is not itself a mobility/falls outcome; strength/function unchanged)
Blood-flow-restricted resistance training · knee-extensor strength	1 exploratory RCT (*n* = 22) + 2 cases	RCT	Between-group difference 15.0% (per-protocol, *p* = 0.026) favoring BFR (preserved vs. −9% control); IBM-FRS favored BFR	12 wk	Low	↓ risk of bias (exploratory, per-protocol result); ↓ imprecision
Blood-flow-restricted training · harms	1 RCT (*n* = 22)	RCT	2 intervention-related dropouts (severe fatigue; a fall)	12 wk	Very low	Very small; imprecise
Orthoses (stance-control) · knee stability/gait	1 uncontrolled study (*n* = 9)	Non-randomized	Feasibility/biomechanical signal; no controlled functional outcome	~6 months (within-subject)	Very low	Non-randomized, uncontrolled, small
Robotic assistance (HAL) · ambulation (2 min walk)	1 crossover RCT (mixed cohort; IBM subset) + case series (*n* = 3)	RCT/series	2MWT +10.1% (95% CI 0.7–19.5), *p* = 0.037 in mixed cohort	Course-based	Very low	↓ indirectness (mixed neuromuscular cohort); ↓ imprecision; ↓ risk of bias
**(c)**						
**Question · Outcome**	**Studies (Participants)**	**Design**	**Estimate**	**Certainty**		**Reasons**
A1 · Rate of lower-limb (quadriceps) strength decline	~11 cohorts (*n* ≈ 10–140 each)	Prognostic cohort	Quantitative knee-extension loss ~9–28%/yr; quantitative testing consistently far more sensitive than MMT	Moderate		Large, consistent and reproducible quantitative-over-MMT sensitivity effect across ~11 cohorts; downgraded once for between-study heterogeneity in measures and rates (not upgraded).
A1 · Functional decline (IBM-FRS) and progression to gait-aid/wheelchair dependence	~6 cohorts	Prognostic cohort	IBM-FRS ~6%/yr; independent walking commonly lost by ~7 yr; wheelchair by median ~14 yr	Low		↓ risk of bias; ↓ inconsistency
A2 · Lower-limb (quadriceps) strength predicts gait and transitional-task performance (association)	~6–8 cross-sectional/cohort	Cross-sectional/cohort	Consistent positive associations: knee-extension strength correlates with gait speed, 2MWT/6MWT, TUG and sit-to-stand, and declines as patients move to more supportive gait aids	Low		Observational start; ↓ risk of bias (small, cross-sectional samples); ↓ imprecision
A3 · Prevalence/burden of falls	~4 surveys/cross-sectional (*n* = 8–470)	Cross-sectional	Fall history up to 98%; ≥1 fall/6 mo ~40%; fall-related fracture ~26%	Low		Observational start; ↓ risk of bias (self-report/recall) offset by consistency
A4 · Lower-limb sensory/peripheral-nerve function (thresholds, fiber pathology)	2 cross-sectional (*n* = 9; 19)	Cross-sectional	Large-fiber sensory dysfunction common: abnormal vibratory thresholds; large-fiber neuropathy ~67%; small-fiber skin-biopsy pathology ~72%	Very low		Observational start; ↓ risk of bias (small, uncontrolled/minimally controlled, single-center); ↓ imprecision. The sensory → balance/falls link is not tested.
**(d)**						
**Question**			**Status**			
Measured postural balance/posturography in IBM			No eligible IBM study identified despite dedicated balance/posturography terms in arms A3 and A4			
Proprioceptive acuity (joint-position sense, motion-detection thresholds) in IBM			No eligible IBM study identified despite dedicated terms in arm A4			
Task-specific/transfer-focused rehabilitation as an intervention			No eligible IBM trial identified desoute dedicated A5a rehabilitation search terms.			
Dedicated balance training as an intervention			No eligible IBM trial identified despite dedicated A5a (training) and A3 (balance) search terms.			

## Data Availability

All data supporting the findings of this review are contained within the article, its tables, and Appendix A. The full extraction dataset and the risk-of-bias workbook are available from the corresponding author on reasonable request.

## Supplementary Materials

The following supporting information can be downloaded at https://www.mdpi.com/article/10.3390/jcm15166304/s1: Supplementary File S1—risk_of_bias_workbook. Supplementary File S2—PRISMA_checklist. Supplementary File S3—fulltext_exclusions. Supplementary File S4—search_strategies. Supplementary File S5—companion_report_map. Supplementary File S6—study_disposition.

## Abbreviations

The following abbreviations are used in this manuscript:

IBM/sIBM	(sporadic) inclusion body myositis
IBM-FRS	Inclusion Body Myositis Functional Rating Scale
MMT	manual muscle testing
QMT/MVICT	quantitative/maximal voluntary isometric contraction testing
TUG	timed up-and-go
2MWT/6MWT	two-/six-minute walk test
BFR	blood-flow-restricted resistance training
HAL	Hybrid Assistive Limb
IVIg/SCIg	intravenous/subcutaneous immunoglobulin
ADL	activities of daily living
GRADE	Grading of Recommendations, Assessment, Development and Evaluations
PRISMA	Preferred Reporting Items for Systematic Reviews and Meta-Analyses
SWiM	Synthesis Without Meta-analysis
RoB 2	Cochrane Risk of Bias tool 2
ROBINS-I	Risk Of Bias In Non-randomized Studies of Interventions
QUIPS	Quality In Prognosis Studies
JBI	Joanna Briggs Institute critical-appraisal checklists
MRC	Medical Research Council (muscle-strength grading)
MVIC	maximum voluntary isometric contraction
6MWD	six-minute walk distance
DEXA	dual-energy X-ray absorptiometry
FES-I	Falls Efficacy Scale–International
KLRG1	killer-cell lectin-like receptor G1

## Appendix A. Search Strategy by Arm, with Records Identified and Studies Included

All searching was title/abstract/keyword free-text (no controlled vocabulary relied upon), with each concept block combined with the IBM population block (A0) using AND. Blocks are shown in PubMed syntax; the same terms were translated to Embase/CENTRAL (:ti,ab,kw), Scopus/TITLE-ABS-KEY, Web of Science/TS=, and EBSCO (CINAHL, SPORTDiscus) TI()/AB(), and PEDro and the trial registries (ClinicalTrials.gov, WHO ICTRP) were searched with the population and intervention terms. Record counts shown are the per-arm records identified in seven databases—PubMed, Embase, Scopus, Web of Science, Cochrane CENTRAL, CINAHL, and SPORTDiscus (PubMed and Scopus run on 28 July 2026; the remainder run with the same strings); PEDro and the trial registries were screened directly and are not tabulated as arm counts. The full multi-database set was exported and deduplicated (records → reports → studies) before screening, and the “Included” column gives the final number of unique IBM studies contributing to each review question after screening and study-level de-duplication.

### Appendix A.1. Core Direct-IBM Search Arms (Counted Evidence Base)

A0—IBM population block (reused in every arm): “inclusion body myositis” OR “sporadic inclusion body myositis” OR “inclusion body myopathy” OR sIBM *Reference—total IBM literature (population block alone): PubMed 2680.*

A1—Natural history/strength progression. Concept terms: strength OR weak* OR dynamomet* OR MVIC OR “quantitative muscle testing” OR QMT OR “manual muscle testing” OR MMT OR “IBM-FRS” OR progression OR longitudinal OR prognos* OR “natural history” OR “functional decline”. *Records identified:* PubMed 957, Embase 2120, Scopus 1695, Web of Science 1337, CENTRAL 118, CINAHL 182, SPORTDiscus 14 (arm total: 6423). *Included studies:* 16.

A2—Motor performance (gait and transitional tasks). Concept terms: gait OR walk* OR ambulat* OR kinematic* OR kinetic* OR “sit-to-stand” OR “sit to stand” OR “stand-to-sit” OR “chair rise” OR “timed up and go” OR TUG OR “six-minute walk” OR “6-min walk” OR “2-min walk” OR 6MWT OR 2MWT OR stance OR “swing phase” OR cadence OR “gait speed”. *Records identified:* PubMed 113, Embase 386, Scopus 247, Web of Science 170, CENTRAL 50, CINAHL 29, SPORTDiscus 5 (arm total: 1000). *Included studies:* 10.

A3—Falls and balance. Concept terms: fall OR falls OR falling OR faller* OR balance OR “postural sway” OR sway OR stability OR instability OR “fear of falling”. *Records identified:* PubMed 75, Embase 186, Scopus 152, Web of Science 134, CENTRAL 22, CINAHL 18, SPORTDiscus 5 (arm total: 592). *Included studies:* 7.

#### Arm A4—Lower-Limb Sensory, Proprioceptive & Neurological Function

Review question A4 combines the IBM population block (A0) with a proprioceptive/sensory/neurological concept block to test directly whether IBM evidence exists on lower-limb sensory, proprioceptive, and neurogenic function: propriocept* OR “joint position sense” OR “position sense” OR “movement sense” OR kinesth* OR kinaesth* OR “sensory deficit” OR “sensory loss” OR “sensory neuropathy” OR “sensory function” OR “sensory nerve” OR “vibration sense” OR “vibratory sense” OR “plantar sensation” OR sensorimotor OR posturograph* OR “postural sway” OR neurogenic OR neuropath* OR polyneuropath* OR “nerve conduction” OR “large fibre” OR “large fiber” OR “motor unit” OR denervation OR reinnervation OR “peripheral nerve” OR “nerve hypertrophy” OR electromyograph* OR EMG (Scopus run in two halves to avoid a wildcard-expansion failure).

**Table A1:** Arm A4—Records identified per database.

Database	Records
Scopus	944
Embase	706
Web of Science	470
CINAHL	43
Cochrane CENTRAL	13
PubMed	10
SPORTDiscus	3
Raw total (with overlap)	2189
Unique after de-duplication	1399
IBM-specific (mention IBM/sIBM)	898

Screening outcome. Two studies that *directly measured sensory function* in sporadic IBM met eligibility and were added to the counted evidence base as review question A4 (Arnardottir, Svanborg & Borg [39]—quantitative somatosensory thresholds; Sturm et al. [40]—multimodal small- and large-fiber assessment), taking the base from 65 to 67. The remaining outputs—motor electromyography/motor-unit and neurogenic studies (Soltanzadeh, Nojszewska, Mano, Elmansy, Abualhayjaa, Lee), diagnostic-utility-only reports, non-sporadic-IBM (familial/VCP) and other-disease records, and a concordant secondary systematic review (Jeon et al. [6])—did not meet the counted-evidence criteria and are retained as mechanistic context in Discussion (Section 4.2), keeping motor neurophysiology separate from sensory function. Crucially, no IBM study measured proprioceptive acuity (joint-position sense) or posturographic postural sway, so those specific outcomes remain searched-but-empty gaps (Table 4d).

A5a—Intervention effects (non-pharmacological). Concept terms: exercise OR resistance OR aerobic OR “blood flow restriction” OR BFR OR rehab* OR “physical therapy” OR physiotherap* OR training OR orthos* OR orthotic* OR exoskeleton* OR robot* OR HAL OR “hybrid assistive limb”. *Records identified:* PubMed 148, Embase 323, Scopus 410, Web of Science 304, CENTRAL 39, CINAHL 46, SPORTDiscus 10 (arm total: 1280).

A5b—Intervention effects (pharmacological). Concept terms: immunoglobulin* OR IVIG OR corticosteroid* OR prednis* OR bimagrumab OR sirolimus OR rapamycin OR arimoclomol OR “monoclonal antibody” OR immunosuppress* OR “drug therapy”. *Records identified:* PubMed 399, Embase 875, Scopus 1228, Web of Science 744, CENTRAL 87, CINAHL 82, and SPORTDiscus 1 (arm total: 3416).

A5—Intervention effects (combined, A5a + A5b). *Included studies:* 40 (pharmacological and non-pharmacological streams detailed in Table 2a–d; certainty in Table 4a,b).

**Table A2:** Records identified per arm and database. PubMed and Scopus were run on 28 July 2026; Embase, Web of Science, Cochrane CENTRAL, CINAHL, and SPORTDiscus were run with the same strings. PEDro and the trial registries (ClinicalTrials.gov, WHO ICTRP) were searched with the population/intervention terms and screened directly, and are not tabulated as arm counts. Counts are records identified before de-duplication (arms and databases overlap heavily).

Arm	PubMed	Embase	Scopus	Web of Science	CENTRAL	CINAHL	SPORTDiscus	Arm Total (Raw)	Included
A1	957	2120	1695	1337	118	182	14	6423	16
A2	113	386	247	170	50	29	5	1000	10
A3	75	186	152	134	22	18	5	592	7
A5a	148	323	410	304	39	46	10	1280	—
A5b	399	875	1228	744	87	82	1	3416	—
A5 (combined)	—	—	—	—	—	—	—	—	40
All arms (raw, with overlap)	1692	3890	3732	2689	316	357	35	12,711	65

Across the seven databases, the arms returned 12,711 records before de-duplication; because the arms and databases overlap heavily (a study may appear in several arms and in several databases), the unique record set is far smaller. Within-database de-duplicated unions were 1240 (PubMed) and 2427 (Scopus); the corresponding within-database unions for the other five databases were not separately recorded. All result sets were exported into one reference manager and de-duplicated (records → reports → studies) before two-reviewer screening. These arms yielded 65 studies; with the two studies from the sensory arm A4 (Appendix A.1, Table A1), 67 studies are included in total (58 full reports; nine conference abstracts). Studies sum to more than the review-question totals because a study may inform more than one question (16 + 10 + 7 + 2 + 40 counts each contribution). The full-text exclusion list and the study-level reconciliation of companion reports are provided in the accompanying protocol appendices.

### Appendix A.2. Provenance of the Indirect and Mechanistic Material (Discussion Only)

The non-IBM, mechanistic, and comparative literature cited in the Section 4 forms no part of the counted evidence base and contributed no study to any GRADE profile. It was assembled from three sources: (i) the previous version’s discussion of mechanistic, electrodiagnostic, and related reports, together with the systematic reviews identified in the previous search; (ii) peer suggestions—references recommended during peer review (Garay-Sánchez et al.; Echemendía del Valle et al.; Bissolotti et al.), cited only as indirect context; and (iii) a supplementary non-IBM search conducted for the previous version of this review, restricted to populations with non-IBM muscular weakness, which addressed the two areas where direct IBM evidence was sparsest—assistive-device use and falls, and proprioceptive deficits. The supplementary search strings and record counts are reported below for transparency; none of these records were added to the counted IBM evidence base, and all of this material is used solely as clearly labeled context for developing hypotheses.

Supplementary search 1—Assistive-device use and falls in non-IBM lower-limb weakness (PubMed; previous version). Terms: (“assistive device”[tiab] OR “walking aid”[tiab]) AND (“gait”[tiab] OR “fall*”[tiab]) AND (“increased risk”[tiab] OR “increased rate”[tiab] OR “increased fear”[tiab] OR “cadence”[tiab] OR “cycl*”[tiab] OR “speed”[tiab] OR “kinematics”[tiab] OR “step”[tiab]). Records identified: 271 → screened: 266 → full text assessed: 38 → 2 non-IBM studies retained as Discussion context.

Supplementary search 2—Proprioceptive deficits in non-IBM lower-limb weakness (PubMed; previous version). Terms: (“lower-extremity”[tiab] OR “ankle*”[tiab] OR “knee*”[tiab]) AND (“weak*”[tiab]) AND (“propriocep*”[tiab]) AND (“deficit”[tiab] OR “threshold”[tiab] OR “sway”[tiab]). Records identified: 37 → screened: 31 → full text assessed: 6 → non-IBM proprioception/postural-control studies retained as Discussion context.
