# Supplementary material for: Lower-Limb Motor Function, Mobility, Balance, Falls and Intervention Effects in Inclusion Body Myositis: A Systematic Review
Source: J Clin Med. 2026 Aug 14;15(16):6304. doi: 10.3390/jcm15166304 (PMC13513426; doi:10.3390/jcm15166304)
Supplement: Supplementary file 1 [file jcm-15-06304-s001.zip › S2_PRISMA_checklist.pdf]

Supplementary File S2. PRISMA 2020 Checklist

Manuscript: Lower-limb motor function, mobility, balance, falls and intervention effects in inclusion body myositis: a systematic review.

Item locations refer to the section of the main manuscript in which each item is reported. This review is a qualitative (narrative) synthesis without meta-analysis; items specific to meta-analysis and to automation tools are marked "Not applicable".

Title

#	Checklist item	Location / reported
1	Identify the report as a systematic review.	Title

Abstract

#	Checklist item	Location / reported
2	See the PRISMA 2020 for Abstracts checklist (structured abstract).	Abstract (Background/Objectives; Methods; Results; Conclusions)

Introduction

#	Checklist item	Location / reported
3	Describe the rationale for the review in the context of existing knowledge.	Section 1 (Introduction)
4	Provide an explicit statement of the objective(s) or question(s).	Section 1 (five review questions A1–A5)

Methods

#	Checklist item	Location / reported
5	Specify the inclusion and exclusion criteria and how studies were grouped.	Section 2.3 (Eligibility criteria)
6	Specify all databases, registers and other sources searched; date last searched.	Section 2.4; Appendix A; Supplementary File S4
7	Present the full search strategies for all sources.	Appendix A; Supplementary File S4
8	Specify the methods to decide whether a study met the inclusion criteria (reviewers, independence).	Section 2.5 (two independent reviewers; disagreements resolved by consensus between them; for full-text screening, reviewers blinded to bibliographic data and records presented in randomized order)
9	Specify the methods to collect	Section 2.6 (Data extraction)

#	Checklist item	Location / reported
	data from reports (reviewers, independence).	
10a	List and define all outcomes for which data were sought.	Sections 2.3, 2.6
10b	List and define all other variables for which data were sought.	Section 2.6
11	Specify the methods used to assess risk of bias, including tools and independence.	Section 2.7; Supplementary File S1 (RoB workbook)
12	Specify the effect measure(s) used in synthesis or presentation of results.	Sections 2.6, 2.9 (direction, magnitude, precision; effect estimates with 95% CI where reported)
13a	Describe the processes used to decide which studies were eligible for synthesis.	Section 2.9 (grouping by review question and outcome domain)
13b	Describe methods to prepare data for presentation/synthesis.	Sections 2.6, 2.9
13c	Describe methods used to tabulate/visually display results.	Section 2.9; Tables 1–4; Figures 1–4
13d	Describe methods used to synthesize results; rationale.	Section 2.9 (structured narrative synthesis following SWiM; meta-analysis not appropriate)
13e	Describe methods to explore heterogeneity.	Section 2.9 (clinical/methodological heterogeneity; effect-oriented narrative)
13f	Describe sensitivity analyses.	Not applicable (no quantitative pooling)
14	Describe methods to assess risk of bias due to missing results (reporting bias).	Section 2.8 (publication-bias domain within GRADE)
15	Describe methods to assess certainty in the body of evidence.	Section 2.8 (GRADE, adapted for prognostic questions)

Results

#	Checklist item	Location / reported
16a	Describe results of the search and selection (numbers of records/reports).	Section 3 opening; Figure 1 (PRISMA flow)
16b	Cite studies excluded at full text	Supplementary File S3 (full-text exclusion

#	Checklist item	Location / reported
	and give reasons.	list)
17	Cite each included study and present its characteristics.	Table 1; Tables 2a–2c; References
18	Present risk-of-bias assessments for each included study.	Tables 3a–3f; Supplementary File S1
19	Present results of all outcomes (summary statistics, estimates, precision).	Sections 3.1–3.5; Tables 1, 2a–2c, 4a–4d
20a	For each synthesis, summarize characteristics/risk of bias of contributing studies.	Sections 3.1–3.5; Tables 3a–3f
20b	Present results of all statistical syntheses.	Not applicable (narrative synthesis); direction/magnitude/precision given in text and tables
20c	Present results of investigations of heterogeneity.	Sections 3.1–3.5 (contradictory findings examined)
20d	Present results of sensitivity analyses.	Not applicable
21	Present assessments of risk of bias due to missing results.	Section 3.8 (GRADE; publication-bias considerations)
22	Present assessments of certainty for each outcome.	Section 3.8; Tables 4a–4d

Discussion

#	Checklist item	Location / reported
23a	Provide a general interpretation of the results in the context of other evidence.	Sections 4.1–4.5
23b	Discuss limitations of the evidence included.	Sections 4.7
23c	Discuss limitations of the review processes used.	Section 4.7
23d	Discuss implications for practice, policy and future research.	Sections 4.4, 4.8

Other information

#	Checklist item	Location / reported
24a	Provide registration information (name of register and registration number) or state not	Section 2.2 (not prospectively registered; no formal dated protocol prepared)

#	Checklist item	Location / reported
	registered.	
24b	Indicate where the protocol can be accessed, or state it was not prepared.	Section 2.2 (no formal dated protocol was prepared)
24c	Describe amendments to protocol information.	Sections 2.2, 4.7 (post-hoc definition of the five-question framework, incl. the A4 arm, and post-hoc reclassification of previously-counted studies to Discussion-only; see Supplementary File S6)
25	Describe sources of financial/non-financial support and role of funders.	Funding statement (no external funding)
26	Declare any competing interests.	Conflicts of Interest statement
27	Report availability of data, code and other materials.	Data Availability Statement; Supplementary Files S1–S6
