# Supplementary material for: Lower-Limb Motor Function, Mobility, Balance, Falls and Intervention Effects in Inclusion Body Myositis: A Systematic Review
Source: J Clin Med. 2026 Aug 14;15(16):6304. doi: 10.3390/jcm15166304 (PMC13513426; doi:10.3390/jcm15166304)
Supplement: Supplementary file 1 [file jcm-15-06304-s001.zip › S3_fulltext_exclusions.pdf]

Supplementary File S3. Reports excluded at full-text assessment, with reasons

Manuscript: Lower-limb motor function, mobility, balance, falls and intervention effects in inclusion body myositis: a systematic review.

This file corresponds to the full-text-eligibility stage of the PRISMA 2020 flow diagram (Figure 1). Of 87 records taken forward for full-text screening, 7 were not retrieved or were of an ineligible report type (Table S3.1), and 80 were assessed for eligibility, of which 16 were excluded with reasons (Table S3.2), leaving 64 studies included (56 full reports and 8 conference abstracts). Reasons are given at the report level; where several reports belonged to one study, they were collapsed to the parent study before counting (study-level de-duplication, Section 2.5).

Table S3.1. Reports sought for retrieval but not retrieved or of an ineligible report type (n = 7)

#	Report	Reason not carried to full-text assessment
1	Salvarani C, Boiardi L, Maldini MC, et al. High-dose immunoglobulin therapy in a case of inclusion-body myositis. *J. Rheumatol.* 1993.	No retrievable full text or abstract.
2	Hirschmann M, Lochmüller H, Toepfer M, et al. High-dose intravenous immunoglobulin (IVIG) in the treatment of inclusion body myositis: a double-blind, placebo-controlled study. *Neurology* 1998 (conference record).	No retrievable full text; conference record superseded by the indexed full trial report (Walter et al., 2000).
3	Cherin P, Eymard B, Laforet P, et al. Treatment of inclusion-body myositis with the association of corticosteroids and intravenous immunoglobulin. *Arthritis Rheum.* 1998.	No retrievable full text or abstract.
4	Cherin P, Pelletier S, Teixeira A, et al. Treatment of inclusion-body myositis with the association of corticosteroids and intravenous immunoglobulin: long-term follow-up. *Arthritis Rheum.* 2001.	No retrievable full text or abstract.
5	Mukunda BN, Dileep Kumar P, Smith HR. Long-lasting effectiveness of intravenous immunoglobulin in a patient with inclusion-body myositis. *Ann. Intern. Med.* 2001. doi:10.7326/0003-4819-134-12-200106190-00031	Letter to the editor (ineligible report type).
6	Landon-Cardinal O, Prince F, Bédard S, et al. Dermoskeletics to preserve mobility and function in	NeuroImage feature, not a primary

#	Report	Reason not carried to full-text assessment
	inclusion body myositis. *Neurology* 2018. doi:10.1212/wnl.0000000000006365	research article (ineligible report type).
7	Smith GC, Moutvic MA, Oh TH. Use of stance-control knee-ankle-foot orthoses in a patient with inclusion body myositis and falls. *Am. J. Phys. Med. Rehabil.* 2014.	Conference poster; abstract only, no retrievable full text.

Table S3.2. Reports assessed at full text and excluded, with reasons (n = 16)

Grouped by the three reason categories shown in the PRISMA flow diagram: out-of-scope outcome (n = 5); biomarker/diagnostic-only or measurement-validation (n = 3); not primary IBM functional data / mixed cohort not isolable (n = 8).

#	Report	Reason for exclusion
1	Ma AK, Dai F, Roy B. In-patient comorbidities in inclusion body myositis: a United States national in-patient sample-based study. 2023.	Out of scope — outcome is the comorbidity profile, not lower-limb motor function, mobility, balance or falls.
2	Price MA, Barghout V, Benveniste O, et al. Mortality and causes of death in patients with sporadic inclusion body myositis. 2016.	Out of scope — outcome is mortality/causes of death, not an in-scope functional outcome.
3	Black C, Cai H, Shabbir A, Wang LH. Relationship between hand function and handheld ultrasound imaging in inclusion body myositis. 2024.	Out of scope — upper-limb/hand function only; no lower-limb/mobility/falls outcome.
4	Archer M, Cooper I, Beer K, et al. A pilot study to evaluate the efficacy of an at-home TheraPutty hand exercise intervention on strength and function in adults with inclusion body myositis. 2026.	Out of scope — hand/upper-limb intervention; no lower-limb/mobility outcome.
5	Alamr M, Pinto MV, Naddaf E. Atypical presentations of inclusion body myositis. 2022.	Out of scope — diagnostic/phenotype description; no in-scope functional outcome reported.
6	Roy B, Rutkove SB, Nowak RJ. Electrical impedance myography as a biomarker of inclusion body myositis: a cross-sectional study. 2020.	Biomarker/diagnostic-only — evaluates a candidate biomarker, not a functional outcome.

#	Report	Reason for exclusion
7	Schütze K, Cooper I, Galna B, et al. Strapped for strength: a comparison study of dynamometry techniques to evaluate knee-extensor strength in inclusion body myositis. 2024.	Measurement-validation study — compares measurement techniques rather than reporting a functional result.
8	Rider LG, Werth VP, Huber AM, et al. Measures of adult and juvenile dermatomyositis, polymyositis, and inclusion body myositis. 2011.	Instrument/measurement review — catalog of outcome instruments, not primary IBM functional data.
9	Raju R, Dalakas MC. Altered gene expression in the muscles of patients with dermatomyositis and sporadic inclusion body myositis before and after therapy with IVIg. 2004.	Not primary IBM functional data — molecular gene-expression study in a mixed DM/sIBM cohort.
10	Helmers SB, Dastmalchi M, Alexanderson H, et al. Limited effects of high-dose IVIG treatment on molecular expression in muscle tissue of patients with inflammatory myopathies. 2007.	Not primary IBM functional data — molecular expression study in a mixed inflammatory-myopathy cohort.
11	Foreman C, Russo P, Davies N, et al. Use of intravenous immunoglobulin therapy for myositis: an audit in South Australian patients. 2017.	Mixed-myositis service audit; IBM-specific lower-limb/functional data could not be isolated.
12	Dalakas MC. Intravenous immunoglobulin in patients with anti-GAD antibody-associated neurological diseases and patients with inflammatory myopathies. 2005.	Mixed/other-disease cohort; IBM-specific in-scope outcome data could not be isolated.
13	Ortlieb A, Olivier J, Bouri M, et al. From gait measurements to design of assistive orthoses for people with neuromuscular diseases. 2015.	Device-design/engineering report across mixed neuromuscular diseases; no IBM-specific patient functional-outcome data.
14	Alexanderson H, Bergegård J, Munters LA, et al. Patients with idiopathic inflammatory myopathies have low muscle endurance rather than low muscle strength. 2009.	Mixed idiopathic-inflammatory-myopathy cohort; IBM-specific in-scope data could not be isolated.
15	Feigean R, Afroun C, Gasnier E, et al. Potential of the MyoSuit, a lightweight wearable lower-limb	Mixed neuromuscular cohort (2 of 4 patients had IBM); device-performance data reported for the group, IBM-

#	Report	Reason for exclusion
	cable-actuated exoskeleton, in patients with neuromuscular disorders (VP.26). 2022.	specific functional data not isolable.
16	Kogawa M, Miura K, Yasuda K, et al. Effect of gait training using Hybrid Assistive Limb on gait ability and overwork weakness in neuromuscular disease. 2020.	Mixed neuromuscular cohort (21 patients; IBM subset not identifiable); IBM-specific functional data could not be isolated.
