# Supplementary material for: Lower-Limb Motor Function, Mobility, Balance, Falls and Intervention Effects in Inclusion Body Myositis: A Systematic Review
Source: J Clin Med. 2026 Aug 14;15(16):6304. doi: 10.3390/jcm15166304 (PMC13513426; doi:10.3390/jcm15166304)
Supplement: Supplementary file 1 [file jcm-15-06304-s001.zip › S4_search_strategies.pdf]

Supplementary File S4. Database-specific search strategies

Manuscript: Lower-limb motor function, mobility, balance, falls and intervention effects in inclusion body myositis: a systematic review.

Overview and reporting notes

The review searched eight bibliographic databases — **MEDLINE/PubMed, Embase, Scopus, Web of Science, Cochrane CENTRAL, CINAHL, SPORTDiscus, and PEDro** — and two trial registries (**ClinicalTrials.gov** and the **WHO ICTRP**); PEDro, which does not support complex Boolean queries, was screened directly. Each review-question concept block was combined with the inclusion body myositis (IBM) population block (A0) using **AND**. Strings are shown in PubMed syntax and were translated to each platform's field-tagged equivalent: Embase/CENTRAL `:ti,ab,kw`, Scopus `TITLE-ABS-KEY( )`, Web of Science `TS=`, and EBSCO (CINAHL, SPORTDiscus) `TI( ) OR AB( )`.

Outcome-direction terms were not used as eligibility filters. After review, terms such as "significant" and "difference" were removed from the intervention/rehabilitation strings so that the search could not preferentially retrieve reports foregrounding statistically significant results, and each string was re-run in all databases on **28 July 2026** (PubMed and Scopus counts below reflect that re-run; the remaining databases were run with the same corrected strings). No date restriction and no language restriction were applied.

A.1 Core direct-IBM search arms

A0 — IBM population block (reused, combined with each concept block using AND):

```
"inclusion body myositis" OR "sporadic inclusion body myositis" OR
"inclusion body myopathy" OR sIBM
```

Reference — total IBM literature (population block alone): PubMed 2,680.

A1 — Natural history / strength progression

```
strength OR weak* OR dynamomet* OR MVIC OR "quantitative muscle
testing" OR QMT OR "manual muscle testing" OR MMT OR "IBM-FRS" OR
progression OR longitudinal OR prognos* OR "natural history" OR
"functional decline"
```

A2 — Motor performance (gait and transitional tasks)

```
gait OR walk* OR ambulat* OR kinematic* OR kinetic* OR "sit-to-stand"
OR "sit to stand" OR "stand-to-sit" OR "chair rise" OR "timed up and
go" OR TUG OR "six-minute walk" OR "6-minute walk" OR "2-minute walk"
OR 6MWT OR 2MWT OR stance OR "swing phase" OR cadence OR "gait speed"
```

A3 — Falls and balance

```
fall OR falls OR falling OR faller* OR balance OR "postural sway" OR
sway OR stability OR instability OR "fear of falling"
```

A4 — Lower-limb sensory, proprioceptive & neurological function (Scopus run in two halves to avoid a wildcard-expansion failure)

```
propriocept* OR "joint position sense" OR "position sense" OR "movement
sense" OR kinesth* OR kinaesth* OR "sensory deficit" OR "sensory loss"
OR "sensory neuropathy" OR "sensory function" OR "sensory nerve" OR
"vibration sense" OR "vibratory sense" OR "plantar sensation" OR
sensorimotor OR posturograph* OR "postural sway" OR neurogenic OR
neuropath* OR polyneuropath* OR "nerve conduction" OR "large fibre" OR
"large fiber" OR "motor unit" OR denervation OR reinnervation OR
"peripheral nerve" OR "nerve hypertrophy" OR electromyograph* OR EMG
```

A5a — Intervention effects (non-pharmacological)

```
exercise OR resistance OR aerobic OR "blood flow restriction" OR BFR OR
rehab* OR "physical therapy" OR physiotherap* OR training OR orthos* OR
orthotic* OR exoskeleton* OR robot* OR HAL OR "hybrid assistive limb"
```

A5b — Intervention effects (pharmacological)

```
immunoglobulin* OR IVIG OR corticosteroid* OR prednis* OR bimagrumab OR
sirolimus OR rapamycin OR arimoclomol OR "monoclonal antibody" OR
immunosuppress* OR "drug therapy"
```

Records identified per arm and database (before de-duplication; re-run 28 July 2026)

Arm	PubMed	Embase	Scopus	Web of Science	CENTRAL	CINAHL	SPORTDiscus	Arm total (raw)	Included
A1	957	2,120	1,695	1,337	118	182	14	6,423	16
A2	113	386	247	170	50	29	5	1,000	9
A3	75	186	152	134	22	18	5	592	6
A4 (sensory)	10	706	944	470	13	43	3	2,189	2
A5a	148	323	410	304	39	46	10	1,280	—
A5b	399	875	1,228	744	87	82	1	3,416	—
A5 (combin	—	—	—	—	—	—	—	—	38

Arm	PubMed	Embase	Scopus	Web of Science	CENTRAL	CINAHL	SPORTDiscus	Arm total (raw)	Included
ed)									
**All arms (raw, with overlap)**	—	—	—	—	—	—	—	**≈15,000**	**64**

Arms and databases overlap heavily, so the unique record set is much smaller than the raw arm totals; all sets were exported into one reference manager and de-duplicated (records → reports → studies) before two-reviewer screening. The main arms (A1–A3, A5) contributed 62 studies and the sensory arm (A4) added 2, giving **64** included studies. The de-duplication cascade and the study-level reconciliation are shown in the PRISMA flow diagram (Figure 1); full-text exclusions are listed in Supplementary File S3.

Registries and PEDro

- **ClinicalTrials.gov** and the **WHO ICTRP** were searched with the population and intervention terms to capture completed, unpublished, and ongoing intervention studies; trial records were linked to eligible publications rather than counted as separate arm totals.
- **PEDro** was screened directly with the population and intervention terms.

Indirect / mechanistic material (Discussion only)

The non-IBM, mechanistic, and comparative literature cited only in the Discussion was **not** part of the counted evidence base and contributed no study to any GRADE profile. It was assembled from three sources: (i) the previous version's Discussion of mechanistic, electrodiagnostic, and related reports, together with the systematic reviews identified in the previous search; (ii) peer suggestions made during review (Garay-Sánchez et al.; Echemendía del Valle et al.; Bissolotti et al.); and (iii) a supplementary non-IBM search conducted for the previous version of this review, in populations with non-IBM muscular weakness, addressing the two areas where direct IBM evidence was sparsest. The previous-version supplementary strings and counts are reported here for transparency:

Supplementary search 1 — Assistive-device use and falls in non-IBM lower-limb weakness (PubMed).

```
("assistive device"[tiab] OR "walking aid"[tiab]) AND ("gait"[tiab] OR
"fall*"[tiab]) AND ("increased risk"[tiab] OR "increased rate"[tiab] OR
"increased fear"[tiab] OR "cadence"[tiab] OR "cycl*"[tiab] OR
"speed"[tiab] OR "kinematics"[tiab] OR "step"[tiab])
```

Records identified 271 → screened 266 → full text assessed 38 → **2 non-IBM studies** retained as Discussion context.

Supplementary search 2 — Proprioceptive deficits in non-IBM lower-limb weakness (PubMed).

```
("lower-extremity"[tiab] OR "ankle*"[tiab] OR "knee*"[tiab]) AND
("weak*"[tiab]) AND ("propriocep*"[tiab]) AND ("deficit"[tiab] OR
"threshold"[tiab] OR "sway"[tiab])
```

Records identified 37 → screened 31 → full text assessed 6 → non-IBM proprioception/postural-control studies retained as Discussion context.

None of these records was added to the counted IBM evidence base or to any GRADE profile.
