# Supplementary material for: Lower-Limb Motor Function, Mobility, Balance, Falls and Intervention Effects in Inclusion Body Myositis: A Systematic Review
Source: J Clin Med. 2026 Aug 14;15(16):6304. doi: 10.3390/jcm15166304 (PMC13513426; doi:10.3390/jcm15166304)
Supplement: Supplementary file 1 [file jcm-15-06304-s001.zip › S5_companion_report_map.pdf]

Supplementary File S5. Study-level de-duplication: companion-report map

Manuscript: Lower-limb motor function, mobility, balance, falls and intervention effects in inclusion body myositis: a systematic review.

The unit of analysis in this review is the **study (or trial), not the publication**. During screening, companion, secondary, and duplicate reports of the same study were collapsed to a single parent study so that no trial or cohort could be counted more than once (Section 2.5); this is the "reports merged" step of the PRISMA 2020 flow diagram (Figure 1). Table S5.1 lists each parent study (as cited in the review, with its reference number) together with the companion/secondary/duplicate reports that were merged into it and the basis for merging. Table S5.2 lists multi-report development programs whose reports were **retained as distinct studies** (because they represent separate trials or phases with non-overlapping primary data) and are flagged so that they are not read as independent replications of a single effect.

Table S5.1. Reports collapsed to a single parent study

Parent study (reference)	Companion / secondary / duplicate report(s) merged	Basis for merging
Follistatin gene therapy — Mendell et al. [58]	Two companion reports of the same gene-therapy study (the 2016 conference abstract in *Molecular Therapy* and the later functional-outcomes publication of the same cohort).	Same study/trial; reports describe the same participants and outcomes.
Arimoclomol RCT — Machado et al. [52] (NCT02753530)	Four companion reports of the same trial (secondary/subgroup analyses and registry records).	Same trial; secondary analyses are not independent replications.
Sirolimus (OPTIMISM) RCT — Badrising et al. [56] (EU 2024-514575-17; ACTRN12620001226998; NCT04789070)	Three companion reports of the same trial (protocol and trial-registry records).	Same trial.
Community aerobic training — Wallace et al. [68]	Wallace et al. 2016 conference abstract describing the design and early results of the same study.	Conference abstract superseded by the full report.

Parent study (reference)	Companion / secondary / duplicate report(s) merged	Basis for merging
Physical function and muscle strength (cross-sectional) — Jørgensen et al. [31]	Jørgensen et al. 2016 conference abstract (*Annals of the Rheumatic Diseases*) reporting the same cohort.	Conference abstract superseded by the full report.
Blood-flow-restricted resistance-training RCT — Jørgensen et al. [65]	Immunological/mechanistic substudy of the same randomized trial.	Substudy of the same trial; shares participants, not an independent replication.
Light-load BFR resistance training — Liang et al. [74]	Duplicate 2019 conference-abstract posting of the same study (two abstract versions of identical content).	Duplicate record.
Fall-risk prediction — Hunn S. et al. [35]	GCOM 2024 companion abstract ("Gait analysis and fall prediction") reporting the same Washington University 62-patient cohort.	Companion conference abstract of the same cohort.

These groupings account for the study-level de-duplication summarized in the PRISMA flow diagram (Figure 1, "reports merged"). A small number of additional duplicate conference-abstract postings of already-included studies were removed at the record level and are not listed individually.

Table S5.2. Multi-report programs retained as distinct studies (relationship flagged, not merged)

Development program	Reports retained as separate studies	Why kept separate
Bimagrumab	Amato et al. single-dose proof-of-concept [51]; RESILIENT phase 2b trial (Hanna et al.) [49]; open-label extension (Sivakumar et al.) [50].	Distinct trials/phases with different designs, durations, and non-overlapping primary data; the extension is identified as arising from RESILIENT so it is not read as independent confirmation.
Sirolimus	Proof-of-concept phase 2b trial (Benveniste et al.) [54]; phase III OPTIMISM trial (Badrising et al.) [56].	Separate trials at different development phases; primary data do not overlap.

Where reports from one program are retained separately, the Results and evidence tables note the relationship so that the reports are not interpreted as independent replications of a single clinical effect (Section 2.5; Tables 2a, 2b).
