# Supplementary material for: Lower-Limb Motor Function, Mobility, Balance, Falls and Intervention Effects in Inclusion Body Myositis: A Systematic Review
Source: J Clin Med. 2026 Aug 14;15(16):6304. doi: 10.3390/jcm15166304 (PMC13513426; doi:10.3390/jcm15166304)
Supplement: Supplementary file 1 [file jcm-15-06304-s001.zip › S6_study_disposition.pdf]

Supplementary File S6. Study disposition between the previous search and the revised search

Manuscript: Lower-limb motor function, mobility, balance, falls and intervention effects in inclusion body myositis: a systematic review.

This companion document maps every study in the previous (Round 5) counted evidence base to its disposition in the revised manuscript, and lists the studies newly added. It is provided to make the redesign fully auditable with respect to review identity, double counting, and the mixing of direct and indirect evidence. Round 5's counted set was taken from its Table 1 (risk-of-bias table, entries #1–65, with entries 57/58/60 absent); the revised counted set is the 64 included studies. A small number of entries below are year/label corrections of the same underlying study rather than true additions or removals.

Summary

Disposition	n
Retained as counted evidence	~30
Added to the counted evidence base	~33
Moved from counted evidence to Discussion-only (indirect)	16
Excluded entirely	14
Collapsed into a parent study	3

Table A. Studies added to the counted evidence base (not counted in Round 5)

Study (revised ref.)	Category	Reason for addition
Arnardottir, Svanborg & Borg 2003 [39]	A4 sensory	New lower-limb sensory/peripheral-nerve arm; directly measured somatosensory thresholds in IBM.
Sturm et al. 2025 [40]	A4 sensory	New A4 arm; multimodal small- and large-fiber assessment in IBM.
Rose et al. 2001 [21]	A1 natural history	Prospective natural-history cohort; now within scope after the date limit was removed.
Nandakumar et al. 2026 [36]	A3 falls	Cross-sectional falls/fractures study identified in the re-run

Study (revised ref.)	Category	Reason for addition
		search.
Danckworth et al. 2014 [37]	A3 falls	Falls/fracture risk in myopathies with isolable sIBM data.
Saiki et al. 2023 [38]	A3 falls	Case of IBM presenting after a fall.
Houston et al. 2016 [75]	A1 natural history (abstract)	Baseline natural-history characteristics.
Hunn S. et al. 2024 [35]	A2/A3 falls (abstract)	Fall-risk/FES-I study (62-patient cohort); the GCOM 2024 companion abstract was collapsed into this study (see Supplementary File S5).
Dalakas 1997 [41]; Walter 2000 [42]; Dalakas 2001 [43]; Soueidan 1993 [44]; Amato 1994 [45]; Dobloug 2012 [46]; Cherin 2016 [48]	A5 pharmacological (IVIg/SCIg)	Immunoglobulin trials/series added when the intervention evidence was made comprehensive.
Dalakas 2009 [53] (alemtuzumab); Sivakumar 2020 [50] (bimagrumab extension); Machado 2023 [52] (arimoclomol; replaces Round 5 "Machado 2021"); Machado 2013 [73] (arimoclomol proof-of-concept RCT — full report)	A5 pharmacological	Expanded drug evidence with distinct trials/phases.
Adler 2026 [57] (pioglitazone); Connor 2023 [59] (testosterone); Goel 2022 [60] (ABC008); Pawlitzki 2022 [55] (sirolimus case); Badrising 2025 [56] (sirolimus OPTIMISM); Mendell 2016 [58] (follistatin gene therapy)	A5 pharmacological	Added to cover the full pharmacological landscape.
Recher 2012 [78]; Johannes 1998 [77]; Kenya 2015 [79]	A5 pharmacological	IVIg case reports/series added.
Spector 1997 [61]; D'Alton 2022 [64]	A5 exercise	Resistance-training studies.
Wallace 2019 [68]	A5 exercise	Aerobic-training randomized feasibility trial.
Tani 2023 [72]	A5 rehabilitation	Inpatient rehabilitation-dose

Study (revised ref.)	Category	Reason for addition
		cohort.
Liang 2019 [74]	A5 exercise (abstract)	BFR matched-control pilot conference report.

Table B. Studies moved from the counted evidence base to Discussion-only (indirect context)

Study (Round 5 label → revised ref.)	Type	Reason for move
Butler 2008 [80]	Non-IBM (post-polio; proprioception/sway)	Non-IBM; retained only as mechanistic context.
Hassan 2001 [81]	Non-IBM (knee osteoarthritis)	Non-IBM context.
Liu 2018 → Liu 2024 [82]	Non-IBM (ankle instability)	Non-IBM context (citation updated).
Santos 2008 [83]	Non-IBM (ankle instability)	Non-IBM context.
Sousa 2017 [84]	Non-IBM (ankle instability)	Non-IBM context.
Franz 2021 → Franz 2025 [85]	Non-IBM (plantar sensation, older adults)	Non-IBM context (citation updated).
Liu 2023 [94]	Non-IBM (mobility-device use, older adults)	Non-IBM context.
Roman de Mettelinge 2015 [95]	Non-IBM (walking aids, older adults)	Non-IBM context.
Soltanzadeh 2010 → 2019 [86]	IBM electrodiagnostic	Motor neurophysiology, not a lower-limb functional outcome.
Nojszewska 2018 [87]	IBM electrodiagnostic	Motor neurophysiology.
Mano 2023 → 2024 [88]	IBM electrodiagnostic	Motor neurophysiology.
Elmansy 2022 [89]	IBM imaging/nerve	Mechanistic (nerve hypertrophy).
Abualhayjaa 2024 [90]	IBM neuropathy	Mechanistic.
Lee 2023 → 2020 [91]	IBM neurophysiology	Mechanistic.
Kirou 2023 → 2025 [92]	IBM immunology (dendritic cells)	Disease-activity marker, not sensory/functional.
Jensen 2025 →	IBM (accelerated aging,	Retained as comparative

Study (Round 5 label → revised ref.)	Type	Reason for move
2024 [93]	abstract)	context (sarcopenia framing).

Table C. Studies excluded entirely from the revised review

Study (Round 5 label)	Type	Reason for exclusion
Koo 2019	Animal (rat)	Animal experiments are outside the counted human evidence base.
Kwon 2020	Animal (rat)	Animal experiment.
Naddaf 2022	Narrative review	Secondary literature; not primary evidence.
Anderson 2022	Narrative/literature review	Secondary literature.
Josop 2022	Systematic review	Secondary literature (Jeon 2026 retained only as a Discussion comparator).
Fritz 2015	Systematic review	Secondary literature.
Mou 2023	Systematic review	Secondary literature.
Price 2016	IBM (mortality)	Out of scope — outcome not lower-limb/mobility/falls (full-text exclusion, S3).
Ma 2023	IBM (comorbidities)	Out of scope (full-text exclusion, S3).
Moreira 2021	Non-IBM biomechanics	Non-IBM; not retained even as context.
Farah 2023	Non-IBM controlled trial	Non-IBM; not retained.
Fujita 2019	Non-IBM quasi-experimental	Non-IBM; not retained.
Feigean 2022	Mixed-cohort device (MyoSuit exoskeleton)	Mixed neuromuscular cohort (2 of 4 patients IBM); IBM-specific functional data could not be isolated (full-text exclusion, S3).
Kogawa 2020	Mixed-cohort device (robotic HAL)	Mixed neuromuscular cohort (21 patients; IBM subset not isolable); IBM-specific data could not be isolated (full-text exclusion, S3).

Table D. Reports collapsed to a parent study (companion/duplicate)

Round 5 entry	Collapsed into	Basis
Johnson 2009	Johnson 2007	Companion report of the same home-

Round 5 entry	Collapsed into	Basis
	[62]	exercise study.
"Jensen 2019" (BFR RCT sub-study)	Jørgensen 2018 (BFR) [65]	Immunological/mechanistic sub-study of the same trial (see Supplementary File S5).
Hunn 2024 (GCOM "gait analysis & fall prediction")	Hunn S. 2024 [35] (212P)	Companion conference abstract of the same 62-patient cohort.

Table E. Studies retained through both versions (with any corrections)

Allenbach [16]; Cortese [18]; Hogrel [17]; Cox [19]; Lilleker [23]; Lindberg [22]; Oldroyd [24]; Sangha [20]; Lindgren (2022→2023) [26]; Dunlap (2011→2014) [27]; Lowes [30] and Lowes L.P. [33]; Oh (2007→2010) [32]; Jørgensen 2017 [31]; Bernhardt — gait [28] and orthosis [69]; Davenport (2014→2015) [29]; Hiscock [34]; Benveniste 2011 [25]; Benveniste 2021 [54]; Hanna (RESILIENT) [49]; Amato 2014 [51]; Cherin 2015 [47]; Johnson 2007 [62]; Orsini (2010→2009) [76]; Suzuki (2021→2020) [71]; Nakajima 2021 [70]; Gualano 2010 [66]; Santos 2014 [67]; Jørgensen 2018 — BFR [65].

Notes. Round 5's counted denominator is approximate because its Table 1 omitted entries 57/58/60. Several rows above reflect corrected publication years for the same underlying study rather than a change of study. Full companion-report groupings are given in Supplementary File S5, and full-text exclusions with reasons in Supplementary File S3.

Appendix S6.1. Search terminology used in the previous version (for transparency)

The search strings run for the previous version of this review are reproduced below with their **terminology unchanged**, and with only the **bracketing and syntax errors identified in peer review corrected** — unmatched or duplicated parentheses closed, curly ("smart") quotation marks replaced with straight quotes, missing or lowercase Boolean operators added, platform-mismatched field tags removed, and duplicate term artifacts deleted. The outcome-direction terms ("significant*", "difference*") present in the previous rehabilitation strings are retained here as they were originally run; these were removed in the revised search (Section 2.4). This terminology is also referenced in Section 2.5 of the manuscript and is provided here as part of the supplementary file.

Previous arm — Strength / functional decline

PubMed

```
("Inclusion Body Myositis"[tiab] OR "Sporadic Inclusion Body
Myositis"[tiab]) AND ("strength" OR "weak*" OR "decline" OR
"functional") AND ("dynamometer*" OR "manometer*" OR "manometry" OR
```

"tensiometer*" OR "Maximum Voluntary Isometric Contraction" OR "Isometric" OR "MVIC" OR "MVICT" OR "Quantitative Muscle Testing" OR "QMT" OR "Manual Muscle Testing" OR "Manual Strength Testing" OR "MMT" OR "MST")

Scopus

TITLE-ABS({Inclusion Body Myositis} OR {Sporadic Inclusion Body Myositis}) AND TITLE-ABS-KEY({strength} OR {weak*} OR {decline} OR {functional}) AND TITLE-ABS-KEY({dynamometer*} OR {manometer*} OR {manometry} OR {tensiometer*} OR {Maximum Voluntary Isometric Contraction} OR {Isometric} OR {MVIC} OR {MVICT} OR {Quantitative Muscle Testing} OR {QMT} OR {Manual Muscle Testing} OR {Manual Strength Testing} OR {MMT} OR {MST})

Embase

("Inclusion Body Myositis" OR "Sporadic Inclusion Body Myositis"):ti,ab AND ("strength" OR "weak*" OR "decline" OR "functional") AND ("dynamometer*" OR "manometer*" OR "manometry" OR "tensiometer*" OR "Maximum Voluntary Isometric Contraction" OR "Isometric" OR "MVIC" OR "MVICT" OR "Quantitative Muscle Testing" OR "QMT" OR "Manual Muscle Testing" OR "Manual Strength Testing" OR "MMT" OR "MST")

Previous arm — Falls / assistive-device use

PubMed

("Inclusion Body Myositis"[Title/Abstract] OR "Sporadic Inclusion Body Myositis"[Title/Abstract]) AND ("fall*"[All Fields] OR "assistive*"[All Fields] OR "aid"[All Fields] OR "device*"[All Fields] OR "cane*"[All Fields] OR "walker*"[All Fields] OR "roll*"[All Fields]) AND ("risk"[All Fields] OR "prevalence"[All Fields] OR "incidence"[All Fields] OR "dependence"[All Fields] OR "usage"[All Fields] OR "pattern"[All Fields])

Scopus

TITLE-ABS({Inclusion Body Myositis} OR {Sporadic Inclusion Body Myositis}) AND TITLE-ABS-KEY({fall*} OR {assistive*} OR {aid} OR {device*} OR {cane*} OR {walker*} OR {roll*}) AND TITLE-ABS-KEY({risk} OR {prevalence} OR {incidence} OR {dependence} OR {usage} OR {pattern})

Embase

("Inclusion Body Myositis" OR "Sporadic Inclusion Body Myositis"):ti,ab AND ("fall*" OR "assistive*" OR "aid" OR "device*" OR "cane*" OR "walker*" OR "roll*") AND ("risk" OR "prevalence" OR "incidence" OR "cadence" OR "cycl*" OR "speed" OR "test" OR "dependence" OR "usage" OR "pattern")

Previous arm — Proprioception / balance / neurological

PubMed

("Inclusion Body Myositis"[tiab] OR "Sporadic Inclusion Body Myositis"[tiab]) AND ("proprio*" OR "balance" OR "stability" OR "stead*" OR "footing" OR "nerv*" OR "neuro*" OR "afferent" OR "axon*" OR "innervat*") AND ("deficit" OR "threshold" OR "motion*" OR "EMG" OR "wave*" OR "excit*" OR "potential*")

Scopus

TITLE-ABS({Inclusion Body Myositis} OR {Sporadic Inclusion Body Myositis}) AND TITLE-ABS-KEY({proprio*} OR {balance} OR {stability} OR {stead*} OR {footing} OR {nerv*} OR {neuro*} OR {afferent} OR {axon*} OR {innervat*}) AND TITLE-ABS-KEY({deficit} OR {threshold} OR {motion*} OR {EMG} OR {wave*} OR {excit*} OR {potential*})

Embase

("Inclusion Body Myositis" OR "Sporadic Inclusion Body Myositis"):ti,ab AND ("proprio*" OR "balance" OR "stability" OR "stead*" OR "footing" OR "nerv*" OR "neuro*" OR "afferent" OR "axon*" OR "innervat*") AND ("deficit" OR "threshold" OR "motion*" OR "EMG" OR "wave*" OR "excit*" OR "potential*")

Previous arm — Task-specific coordination (gait, transitions)

PubMed

("Inclusion Body Myositis"[Title/Abstract] OR "Sporadic Inclusion Body Myositis"[Title/Abstract]) AND ("TUG"[All Fields] OR "sit-to-stand"[All Fields] OR "stand-to-sit"[All Fields] OR "gait*"[All Fields] OR "kinematics"[All Fields] OR "position"[All Fields] OR "moment"[All Fields] OR "stance"[All Fields] OR "loading"[All Fields] OR "walk*"[All Fields]) AND ("risk"[All Fields] OR "cadence"[All Fields] OR "cycl*"[All Fields] OR "speed"[All Fields] OR "test"[All Fields] OR "pattern"[All Fields])

Scopus

TITLE-ABS({Inclusion Body Myositis} OR {Sporadic Inclusion Body Myositis}) AND TITLE-ABS-KEY({TUG} OR {sit-to-stand} OR {stand-to-sit} OR {gait*} OR {kinematics} OR {position} OR {moment} OR {stance} OR {loading} OR {walk*}) AND TITLE-ABS-KEY({risk} OR {cadence} OR {cycl*} OR {speed} OR {test} OR {pattern})

Embase

("Inclusion Body Myositis" OR "Sporadic Inclusion Body Myositis"):ti,ab AND ("TUG" OR "sit-to-stand" OR "stand-to-sit" OR "gait*" OR "kinematics" OR "position" OR "moment" OR "stance" OR "loading" OR

```
"walk*") AND ("risk" OR "cadence" OR "cycl*" OR "speed" OR "test" OR
"pattern")
```

Previous arm — Rehabilitation / intervention

The outcome-direction terms ("significant", "difference*") are shown as originally run and were removed in the revised search (Section 2.4).*

PubMed

```
("Inclusion Body Myositis"[tiab] OR "Sporadic Inclusion Body
Myositis"[tiab]) AND ("exercise" OR "resistance*" OR "rehab*" OR
"blood-flow*" OR "vascular*" OR "training" OR "therapy" OR "treatment"
OR "drug" OR "phase") AND ("study" OR "regimen" OR "program" OR
"week*") AND ("strength" OR "IBM-FRS" OR "aerobic*" OR "significant*"
OR "difference*")
```

Scopus

```
TITLE-ABS({Inclusion Body Myositis} OR {Sporadic Inclusion Body
Myositis}) AND TITLE-ABS-KEY({exercise} OR {resistance*} OR {blood-
flow*} OR {training} OR {therapy} OR {treatment} OR {drug} OR {phase})
AND TITLE-ABS-KEY({study} OR {regimen} OR {program} OR {week*}) AND
({strength} OR {IBM-FRS} OR {aerobic*} OR {significant*} OR
{difference*})
```

Embase

```
("Inclusion Body Myositis" OR "Sporadic Inclusion Body Myositis"):ti,ab
AND ("exercise" OR "resistance*" OR "rehab*" OR "blood-flow*" OR
"vascular*" OR "training" OR "therapy" OR "treatment" OR "drug" OR
"phase") AND ("study" OR "regimen" OR "program" OR "week*") AND
("strength" OR "IBM-FRS" OR "aerobic*" OR "significant*" OR
"difference*")
```

Previous supplementary (non-IBM) searches — Discussion context only

Reproduced here for completeness; the same strings and record counts appear in Supplementary File S4.

Assistive-device use and falls in non-IBM lower-limb weakness — PubMed

```
("assistive device"[Title/Abstract] OR "walking aid"[Title/Abstract])
AND ("gait"[Title/Abstract] OR "fall*"[Title/Abstract]) AND ("increased
risk"[Title/Abstract] OR "increased rate"[Title/Abstract] OR "increased
fear"[Title/Abstract] OR "cadence"[Title/Abstract] OR
"cycl*"[Title/Abstract] OR "speed"[Title/Abstract] OR
"kinematics"[Title/Abstract] OR "step"[Title/Abstract])
```

Proprioceptive deficits in non-IBM lower-limb weakness — PubMed

("Lower-extremity"[tiab] OR "ankle*"[tiab] OR "knee*"[tiab]) AND ("weak*"[tiab]) AND ("propriocep*"[tiab]) AND ("deficit"[tiab] OR "threshold"[tiab] OR "sway"[tiab])
